# Cells‐Micropatterning Biomaterials for Immune Activation and Bone Regeneration

**DOI:** 10.1002/advs.202200670

**Published:** 2022-04-28

**Authors:** Bingjun Zhang, Fei Han, Yufeng Wang, Yuhua Sun, Meng Zhang, Xiaopeng Yu, Chen Qin, Hongjian Zhang, Chengtie Wu

**Affiliations:** ^1^ State Key Laboratory of High Performance Ceramics and Superfine Microstructure Shanghai Institute of Ceramics Chinese Academy of Sciences Shanghai 200050 P. R. China; ^2^ Center of Materials Science and Optoelectronics Engineering University of Chinese Academy of Sciences Beijing 100049 P. R. China

**Keywords:** 3D printed bioceramic scaffolds, bone regeneration, immunomodulatory, MSC‐macrophage spatial interaction, multicellular pattern

## Abstract

Natural tissues are composed of ordered architectural organizations of multiple tissue cells. The spatial distribution of cells is crucial for directing cellular behavior and maintaining tissue homeostasis and function. Herein, an artificial bone bioceramic scaffold with star‐, Tai Chi‐, or interlacing‐shaped multicellular patterns is constructed. The “cross‐talk” between mesenchymal stem cells (MSCs) and macrophages can be effectively manipulated by altering the spatial distribution of two kinds of cells in the scaffolds, thus achieving controllable modulation of the scaffold‐mediated osteo‐immune responses. Compared with other multicellular patterns, the Tai Chi pattern with a 2:1 ratio of MSCs to macrophages is more effective in activating anti‐inflammatory M2 macrophages, improving MSCs osteogenic differentiation, and accelerating new bone formation in vivo. In brief, the Tai Chi pattern generates a more favorable osteo‐immune environment for bone regeneration, exhibiting enhanced immunomodulation and osteogenesis, which may be associated with the activation of BMP‐Smad, Oncostatin M (OSM), and Wnt/β‐catenin signaling pathways in MSCs mediated by macrophage‐derived paracrine signaling mediators. The study suggests that the manipulation of cell distribution to improve tissue formation is a feasible approach that can offer new insights for the design of tissue‐engineered bone substitutes with multicellular interactions.

## Graphical abstract

A multicellular coculture scaffold with different cellular patterns is prepared by loading mesenchymal stem cells (MSCs) and macrophages into the corresponding channels of bioceramic scaffolds, realizing spatial “cross‐talk” of multiple tissue cells. The Tai Chi pattern in scaffolds can generate a more favorable osteo‐immune environment to stimulate macrophages M2‐polarization, induce MSCs osteogenic differentiation, and promote bone tissue regeneration in vivo.

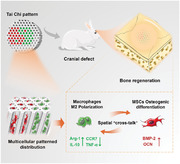

## 1 Introduction

Tissue engineering aims to develop biologically functional substitutes for the purpose of restoring and/or replacing damaged or lost native tissues. One strategy is to incorporate cells within biomaterials to provide a three‐dimensional (3D) microenvironment capable of regulating cell function and ultimately driving new tissue formation. However, in most cases, coculture systems based on tissue‐engineered scaffolds mix different types of cells together in a disordered manner.^[^ ^1^ ^]^ Indeed, various cells in human tissues usually exhibit a certain noncontact spatial arrangement and are organized into complex micropatterns, thus simply mixing cells together does not accurately simulate their state in vivo.^[^ ^2^ ^]^ The spatial distribution of cells and interactions between neighboring cells in native microenvironments are of fundamental importance in determining cell fate, such as migration, proliferation, apoptosis, and differentiation.^[^ ^3^ ^]^ Moreover, the intricate communication among these prepatterned cell populations also plays an essential role in the creation of tissue architecture, the development of organ function, and the maintenance of tissue homeostasis.^[^ ^4^ ^]^ Mimicking the spatial arrangements of these cellular interactions in vitro may allow for the recapitulation of multiple biological processes involved in the formation of natural tissues, resulting in the attainment of higher‐order biological functions and collective behaviors.

Recent synergistic progress in cell biology, biomaterials, and advanced fabrication techniques has enabled spatial control of cell behaviors. Researchers have developed various models to investigate the complex cell–cell interactions using microfluidic,^[^ ^5^ ^]^ 3D bioprinting,^[^ ^6^ ^]^ and “Bottom‐up” modular assembly technologies.^[^ ^7^ ^]^ The result has been the generation of not only tissue‐like biomaterials with bionic cell composition and distribution characteristics but also more life‐like bioengineered organs such as artificial livers,^[^ ^8^ ^]^ intestinal tracts,^[^ ^9^ ^]^ and lungs,^[^ ^6^, ^10^ ^]^ which perfectly recapitulate the multicellular processes in the physiological environment of natural tissues. Despite these encouraging advancements, little attention has been paid to the modulatory effects of diverse cell spatial distributions on multicellular interactions and their consequent implications on tissue repair and regeneration. Consequently, it is quite necessary to develop a tissue‐engineered construct capable of patterning multiple cell types with different spatial arrangements.

The processes of bone formation and fracture healing are accompanied by the 3D regulation of the spatial arrangement and interactions between multiple tissue cells. In the bone microenvironment, bone‐forming cells and immune cells communicate mutually by sharing a variety of signal pathways and cytokine networks to maintain the dynamic balance of bone metabolism.^[^ ^11^ ^]^ Macrophages are the important innate immune cells of the organism. Resident tissue macrophages of bone, termed osteal macrophages, are located immediately adjacent to osteoblasts at sites of active bone remodeling.^[^ ^12^ ^]^ Over 75% of osteoblasts on the diaphyseal endosteal surface are reported to be covered by osteal macrophages like a dome.^[^ ^13^ ^]^ The adjacent spatial location is inextricably linked to their synergistic involvement in bone development and bone biology. Interestingly, there is a significant distinction in the spatial distribution of macrophage subpopulations during tissue repair. As discovered by Troidl et al., M1 macrophages were identified directly adjacent to the collateral vessels during arteriogenesis, while M2 macrophages were located in the most outer perivascular region of the growing collateral vessels.^[^ ^14^ ^]^ Huang et al. also found that macrophages exhibited diverse activation characteristics in different bone regions. Most macrophages adhering to the surface of the cancellous bone surface were in an M1‐like polarization state, whereas M2 cells were predominantly detected in bone marrow or connective tissue. In cortical bone, the majority of these cells did not attach to the bone surface, but rather were scattered throughout the connective tissue.^[^ ^15^ ^]^ This distribution may be related to the functional heterogeneity of macrophages. Most recently, the Michael Angelo research group revealed for the first time the structured spatial distribution of immune cells and tumor cells.^[^ ^16^ ^]^ This was particularly intriguing because this spatial location distribution led to differences in the function of immune cells, demonstrating the diversity of interactions between tumor cells and immune cells within the spatial structure. Inspired by these studies, we hypothesized that different spatial distributions between immune cells and bone‐forming cells might be able to trigger distinct intercellular “cross‐talk”, which would have implications for their differentiation and the formation of bone tissue.

**Figure 1:**
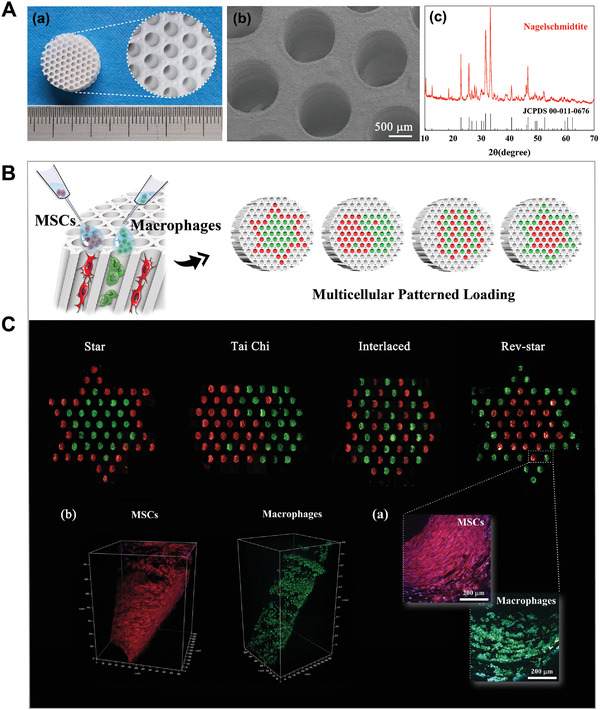
Characterization of the multichannel honeycomb‐like bioceramic scaffolds and patterned spatial configuration of MSCs and macrophages in the scaffolds. A) Optical microscope images (a), typical SEM image (b), and XRD pattern (c) of 3D printed bioceramic scaffolds. B) Schematic illustration of multicellular patterning bioceramic scaffolds. The red channel is loaded with MSCs, and the green channel means it is loaded with macrophages. C) Fluorescence images of MSCs and macrophages arranged in star, Tai Chi, interlaced and reverse‐star (Rev‐star) patterns within the 3D structure of the scaffolds and incubated for 3 days. MSCs and macrophages were labeled with red and green fluorescent probes individually. The 2D (a) and 3D (b) CLSM images of MSCs and macrophages uniformly adhering to the inner walls of their specific channels, showing the patterned spatial distribution of multiple cells in the scaffolds.

Current in vitro studies of immune cell–bone‐forming cell interactions mediated by biomaterials, however, are mostly limited to two‐dimensional (2D) conditioned medium culture or Transwell chamber coculture models, which can only observe the 2D “cross‐talk” between the two kinds of cells. There is still a lack of in‐depth research on the spatial distribution and the interactions of immune cells and bone‐forming cells. In this study, we fabricated a multichannel honeycomb‐like bioceramic scaffold using digital light processing (DLP)‐based 3D printing technology. The honeycomb‐like macropore architecture can serve as a multicellular loading and delivery channel to guide the inward growth of blood vessels and tissues. On this basis, taking advantage of classic bone tissue engineering strategies, different types of cells were selectively loaded into confined channels of specific regions to form multicellular patterns. According to several typical distributions of various cell lineages in the 3D structure of tissues (inside/outside, left/right, or up/down, and interlaced), we constructed four representative complex multicellular patterns in the bioceramic scaffolds, as shown in **Figure** **1**B. Benefiting from the structure design, it could meet the requirement for in vitro monitoring of 3D structural “cross‐talk” between mesenchymal stem cells (MSCs) and macrophages arranged on biomaterials in an ordered manner. On the other hand, by altering the interactions between the two types of cells in the spatial structure (e.g., changes in cell distribution or cell ratio), it was possible to effectively modulate the scaffold‐mediated osteo‐immune response to generate a favorable 3D microenvironment for osteogenesis. To the best of our knowledge, this concept of regulating cell behaviors and functions to promote tissue regeneration by manipulating multicellular patterned distribution has not yet been reported and hence can provide a new perspective on the design of multicellular tissue‐engineered scaffolds. To verify the hypothesis, the interaction between MSCs and macrophages within the spatial structure of scaffolds and the effects of their different structured spatial distributions on the local immune microenvironment induced by scaffolds and bone regeneration in vivo were systematically investigated.

## 2 Result and Discussion

### 2.1 Fabrication of Multicellular Patterning Bioactive Scaffolds

In our research, the nagelschmidtite (NAGEL, Ca_7_Si_2_P_2_O_16_) bioceramic powder was synthesized by a sol–gel process, and honeycomb‐like 3D‐printed bioceramic scaffolds were successfully fabricated. The diameter of channel‐like macropore architecture of the sintered bioceramic scaffolds was maintained at about 1 mm. These tubular channels were oriented at a 30° angle to the vertical direction, as shown in the optical microscopy images in Figure 1A(a), which aids cell attachment. Scanning electron microscope (SEM) images (Figure 1A(b)) showed that abundant micropores were homogeneously distributed on the surface of the NAGEL scaffolds. The porous and loose surface of scaffolds may facilitate fibrin adsorption and cell pseudopod immobilization. The X‐ray diffraction (XRD) analysis indicated that photosensitive resin used in the printing process had been removed by high‐temperature sintering, with no apparent effect on the final crystal phase composition of the NAGEL scaffolds, preserving the pure crystal phase of Ca_7_Si_2_P_2_O_16_ (JCPD 11‐0676) (Figure 1A(c)).

**Figure 2:**
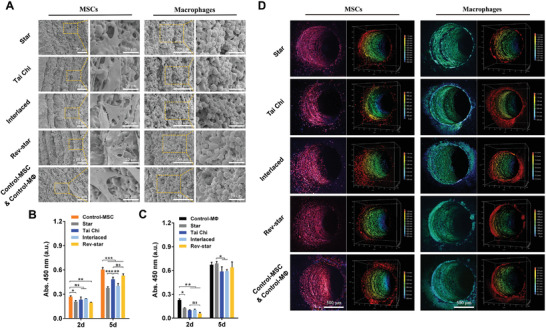
Cell morphology and activity of MSCs and macrophages in different multicellular patterning bioactive scaffolds. A) SEM images of MSCs and macrophages cultured in different multicellular patterning scaffolds at a ratio of 2:1 for 5 days. B) MSCs and C) macrophages proliferation in different multicellular patterning scaffolds for 2 and 5 days, respectively. Data presented as mean ± SD, *n* = 4 for each scaffold type, *p*‐values are calculated using one‐way ANOVA with Tukey correction. * indicates significant difference, *p < 0.05, **p < 0.01, and ***p < 0.001; ns, not statistically significant. D) CLSM 2D (left side) and 3D (right side) images of MSCs and macrophages penetrating deep into channels after incubating for 5 days. MSCs and macrophages were labeled with red and green fluorescent probes, respectively. MSCs or macrophages monocultured in the scaffolds were used as controls and named as Control‐MSC and Control‐MΦ, respectively. The two kinds of cells grew well in these multicellular patterning scaffolds, exhibiting good cell viabilities.

Based on the parallel channel structure of the honeycomb‐like scaffold, we constructed tissue‐engineered bioceramic scaffolds with star‐, Tai Chi‐, interlaced‐, and reverse star‐like multicellular arrangement patterns by loading MSCs and macrophages into different corresponding microchannels, which were referred to as Star, Tai Chi, Interlaced, and Rev‐star in the following discussion (Figure 1B). To visualize the patterned distribution of multiple cells in the scaffolds, MSCs and macrophages were labeled independently with red and green fluorescent probes. After 3 days of incubation, fluorescence imaging of multicellular patterns within the scaffolds was monitored by confocal laser scanning microscopy (CLSM). The results are shown in Figure 1C. It was observed from the surface magnified image (Figure 1C(a)) and the 3D image (Figure 1C(b)) that MSCs and macrophages uniformly attached on the inner walls of their specific channels and penetrated deeply into them. Subsequently, we investigated the viability and proliferation activity of the two types of cells in different cell patterns. SEM and CLSM images showed the adhesion and morphology of MSCs and macrophages cocultured at a 2:1 ratio in bioceramic scaffolds with different multicellular patterns for 5 days. As shown in **Figure** **2**A, MSCs cocultured in all patterns were tightly attached to the scaffolds and spread with an abundance of pseudopodia. Additionally, macrophages from all groups were connected to each other on the inner walls of the scaffold channels and clustered together. Higher magnification images showed that these macrophages had distinctive cell morphologies and protruded filopodia, as well as a lot of protrusion and wrinkled peripheral cytoplasm on the cell surface, indicating that the cells were polarized. It can be seen from the CLSM 2D and 3D images (Figure 2D) that the two types of cells not only permeated into the entire channels, but also had a tendency to migrate outward after incubating for 5 days. cell counting kit‐8 (CCK‐8) assays (Figure 2B,C) determined that MSCs and macrophages in each group expanded significantly with the culture time, demonstrating that the cells grew well in these multicellular patterning scaffolds, exhibiting good cell viabilities.

### 2.2 Multicellular Pattern Directs Macrophage Response In Vitro

**Figure 3:**
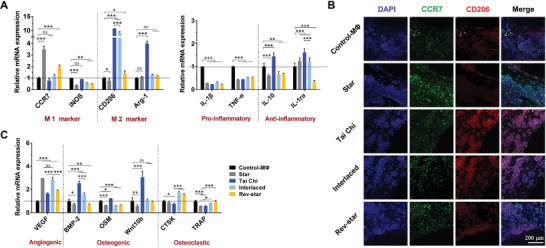
In vitro immune responses of macrophages in different multicellular patterning bioactive scaffolds with a 2:1 ratio of MSCs to macrophages. A) Gene expression of surface markers and pro/anti‐inflammatory cytokines of macrophages in different multicellular patterns on day 3. B) CCR7 (M1 polarized marker, green) and CD206 (M2 polarized marker, red) immunofluorescent staining images of macrophages in different multicellular patterns on day 3. C) Gene expression of regulatory factors associated with tissue repair and remodeling in macrophages grown on the scaffolds with different multicellular patterns for 3 days. Data presented as mean ± SD, *n* = 4 for each scaffold type, *p*‐values are calculated using one‐way ANOVA with Tukey correction, *p < 0.05, **p < 0.01, and ***p < 0.001; ns, not statistically significant. Tai Chi pattern induced the macrophages to differentiate toward a more anti‐inflammatory and pro‐healing extreme phenotype compared with the other multicellular patterns.

The spatial interactions between MSCs and macrophages in different cell arrangement patterns were further investigated. First, to determine the influence of multicellular spatial distribution patterns on macrophage polarization, quantitative real‐time polymerase chain reaction (qRT‐PCR) analysis was performed to detect the expression of genes related to inflammatory regulation in macrophages, including macrophage phenotype markers (chemokine receptor CCR7 (CCR7), inducible nitric oxide synthase (iNOS), macrophage mannose receptor (CD206), and arginase‐1 (Arg‐1)), canonical pro‐inflammatory genes (interleukin‐1β (IL‐1β) and tumour necrosis factor‐α (TNF‐α)), and anti‐inflammatory genes (interleukin‐1 receptor antagonist (IL‐1rα) and interleukin‐10 (IL‐10)). The analysis results showed that when MSCs and macrophages were arranged in a Tai Chi pattern with a 2:1 cell ratio, the gene expression of M2 macrophage markers, CD206 and Arg‐1, as well as anti‐inflammatory cytokines, IL‐1rα and IL‐10, was significantly upregulated compared with the other three multicellular patterns and Control‐MΦ groups after incubating for 3 days. By comparison, genes associated with the M1 macrophage surface marker CCR7 and proinflammatory cytokines (IL‐1β and TNF‐α) exhibited the opposite trend, with relatively decreased expression levels (**Figure** **3**A). Although M1 maker iNOS expression in the Tai Chi pattern group was slightly higher than that in the other pattern groups, it was not significantly different from that of the Control‐MΦ group without cell patterns. In general, the findings suggested that macrophages grown in the Tai Chi pattern tended to differentiate toward a more anti‐inflammatory extreme phenotype. Subsequent immunofluorescent staining of these cells further confirmed the gene expression results. As shown in Figure 3B, more remarkable expression of CD206 signal relative to CCR7 labeled fluorescence signal could be observed in the Tai Chi group, followed by the Interlaced, Rev‐star, and Star groups, suggesting that the Tai Chi pattern was more effective in inducing M2 macrophage activation and reducing M1 macrophage polarization.

Apart from regulating inflammatory environments, macrophages can mediate the interactions among immunomodulation, angiogenesis, and bone regeneration processes through the secretion of regulatory factors relevant to tissue repair and remodeling.^[^ ^17^ ^]^ We therefore examined the gene expression levels of angiogenic, osteogenic, and osteoclastic factors of macrophages in different multicellular patterns to demonstrate the osteo‐immunomodulatory capacity of the multicellular patterning bioceramic scaffolds. It was found that the Tai Chi pattern substantially promoted the expression of osteogenic factor genes in macrophages, including bone morphogenetic protein‐2 (BMP‐2), oncostatin M (OSM), and Wingless‐type MMTV integration site family member 10b (Wnt10b), while inhibiting the gene expression of osteoclastic factors cathepsin K (CTSK) and tartrate‐resistant acid phosphatase (TRAP), as compared with the other multicellular patterns. Moreover, the Tai Chi group exhibited a much higher expression level of the vascular endothelial growth factor (VEGF) than the unpatterned Control‐MΦ group (Figure 3C). Briefly, macrophages in the Tai Chi pattern group generated a more favorable osteo‐immune microenvironment for osteogenesis. These results corroborated the regulatory role of MSC‐macrophage patterned spatial distribution in the scaffold‐mediated osteo‐immune responses of macrophages.

### 2.3 Multicellular Pattern Promotes MSCs Differentiation In Vitro

**Figure 4:**
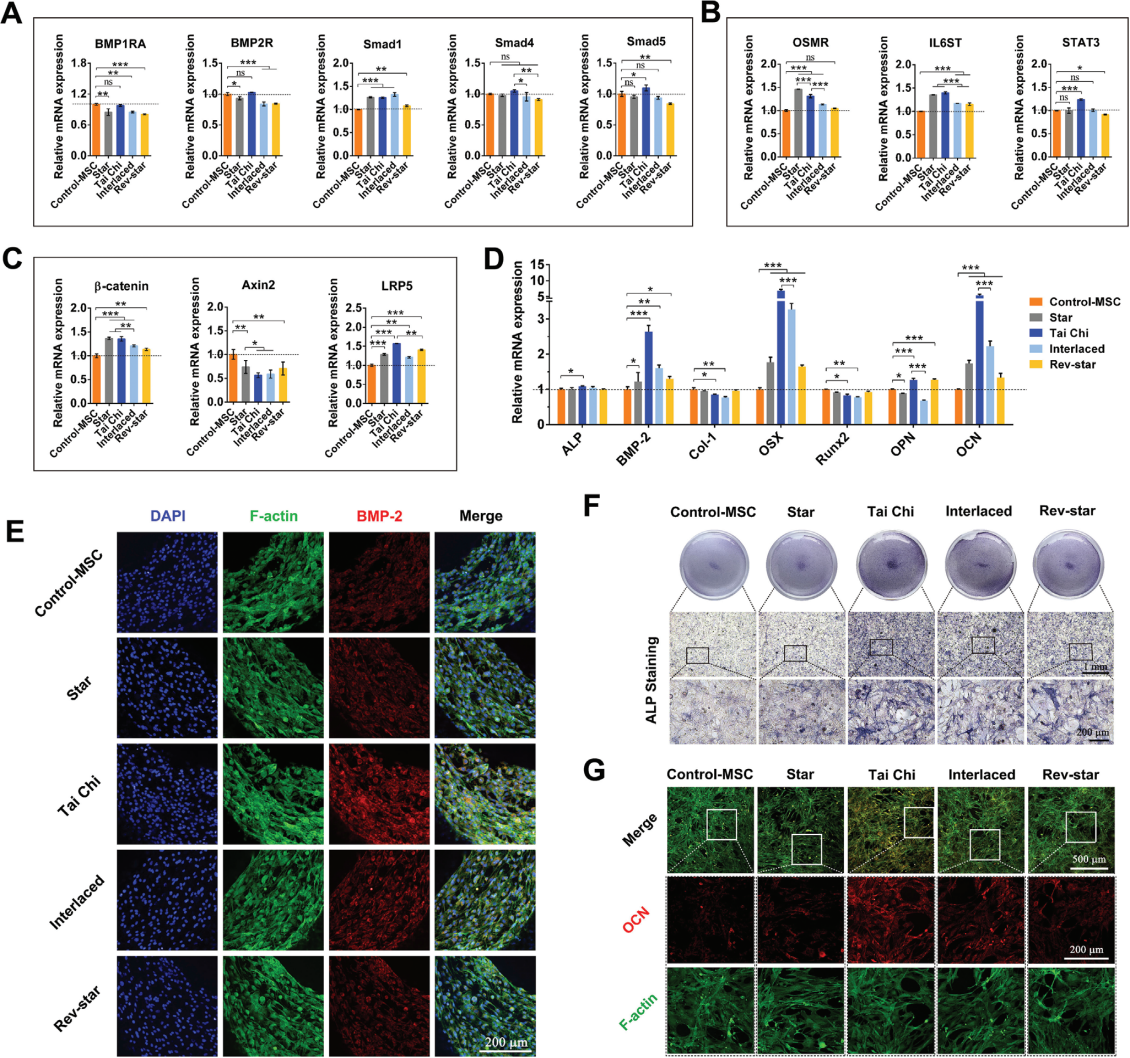
In vitro osteogenic differentiation behaviors of MSCs in different multicellular patterning bioactive scaffolds with a 2:1 ratio of MSCs to macrophages. A–C) Gene expression of BMP‐Smad, OSM, and Wnt/β‐catenin signaling pathway‐associated mediators in MSCs cultured on different multicellular patterning scaffolds for 3 days. D) The osteogenic gene expression of MSCs in different multicellular patterns on day 3. Data presented as mean ± SD, *n* = 4 for each scaffold type, *p*‐values are calculated using one‐way ANOVA with Tukey correction, *p < 0.05, **p < 0.01, and ***p < 0.001; ns, not statistically significant. E) Representative immunofluorescence images of BMP‐2 protein expression in MSCs grown on scaffolds with different multicellular patterns for 3 days. F) ALP staining and G) immunofluorescent staining of OCN for MSCs cultured on scaffolds with different multicellular patterns for 7 days. The Tai Chi pattern with a 2:1 ratio of MSCs to macrophages could significantly promote the osteogenic differentiation of MSCs as compared with the other multicellular patterns.

In response to different multicellular patterns, macrophages may generate different osteo‐immune environments, which in turn may favor or hinder the osteogenic differentiation of MSCs under certain circumstances. Next, to verify this, the effect of multicellular patterns on the differentiation ability of MSCs was investigated. As mentioned earlier, the MSC‐macrophage patterned arrangement affected the expression of BMP‐2, OSM, and Wnt10b genes in macrophages. These osteogenic factors, acting as osteo‐immunomodulators, have been shown to activate the BMP‐Smad, OSM, and Wnt/β‐catenin signaling pathways in MSCs.^[^ ^18^ ^]^ In light of this, we evaluated the expression of the three osteogenic pathway‐related mediators in MSCs grown on different multicellular patterning scaffolds. The results indicated that when the cell ratio of MSCs to macrophages was 2:1, the Tai Chi pattern promoted the up‐regulation of gene expression of BMP pathway‐related components mothers against decapentaplegic homolog 1 (Smad1), Smad4, and Smad5 in MSCs. Although the expression levels of BMP receptor type IA (BMP1RA) and BMP receptor type II (BMP2R) were not significantly elevated as compared to the Control‐MSC group, they were higher than those in the Star, Interlaced, and Rev‐star groups (**Figure** **4**A). Meanwhile, the expression levels of the OSM pathway‐related components oncostatin M Receptor (OSMR), interleukin‐6 signal transducer (IL6ST), and signal transducer and activator of transcription 3 (STAT3) increased dramatically in response to Tai Chi pattern stimulation, indicating that the OSM pathway was activated (Figure 4B). As for key mediators of the canonical Wnt pathway cascade, the gene expression of negative feedback regulator Axin2 was inhibited, while that of β‐catenin and Wnt‐activated coreceptor low‐density lipoprotein receptor‐related protein 5 (LRP5) was increased in the Tai Chi group, demonstrating the positive effect of the Tai Chi pattern on the regulation of the Wnt/β‐catenin pathway (Figure 4C). Additionally, the analysis of osteogenesis‐related gene expression in MSCs revealed that after 3 days of coculture pattern stimulation, the Tai Chi group had significantly higher expression of alkaline phosphatase (ALP), BMP‐2, osterix (OSX), osteopontin (OPN), and osteocalcin (OCN) genes than the Control‐MSC and the other three multicellular pattern groups, indicating a relatively stronger osteogenic potential (Figure 4D). Similar trends were observed for osteogenic marker protein expression in MSCs. A much stronger BMP‐2 marker red fluorescence signal was detected in the Tai Chi pattern group as compared with others (Figure 4E). Besides, Figure 4F,G shows the ALP and OCN staining images of MSCs incubated in different multicellular patterning scaffolds for 7 days. As demonstrated, the two types of staining results followed a consistent trend, with MSCs in the Tai Chi group exhibiting the deepest ALP staining and the highest levels of OCN expression, followed by the Interlaced, Rev‐star, and Star groups, which confirmed that the Tai Chi pattern with a 2:1 ratio of MSCs to macrophages in the scaffolds promoted the osteogenic differentiation of MSCs. From this we hypothesized that the improved osteogenic performance of MSCs in the Tai Chi group might be associated with the activation of BMP‐Smad, OSM, and Wnt/β‐catenin signaling pathways mediated by macrophage‐derived paracrine signaling mediators (OSM, BMP‐2, and Wnt10b).

Next, to further examine the potential molecular mechanism, we collected the supernatants of MSCs and macrophages cultured on scaffolds at day 7 and detected the secretion of their associated cytokines/growth factors by using enzyme‐linked immunosorbent assay (ELISA). As shown in Figure S2 (Supporting Information), the cells in the Tai Chi group secreted lower amounts of proinflammatory cytokines (TNF‐α and iNOS) but more amounts of anti‐inflammatory cytokines (IL‐10 and Arg‐1), which would help to induce the MSCs osteogenic differentiation.^[^ ^19^ ^]^ Moreover, BMP‐2 and OSM concentration in Tai Chi group was also significantly higher than in the other scaffold groups. In accordance with the above gene expression results, these BMP‐2 bound to the BMP1RA and BMP2R receptors on the cell membrane surface of MSCs, activating Smads, which then translated extracellular signals directly into the nucleus, thereby stimulating osteoblast‐specific transcription factors Runt‐related transcription factor 2 (Runx2) and/or OSX and mediating osteogenesis‐related gene transcription in MSCs. Concurrently, the Tai Chi pattern‐modulated upregulation of OSM expression in macrophages activated the surface receptors IL6ST and OSMR of MSCs and induced MSCs differentiation via OSMR/IL6ST heterodimer‐mediated signal transmission,^[^ ^18^, ^20^ ^]^ thus potentiating osteogenesis synergistically with the BMP‐Smad signaling pathway. Additionally, the Tai Chi pattern in the scaffolds improved the expression of Wnt10b, one of the ligands of the canonical Wnt/β‐catenin signaling pathway, which exerted a critical role in inducing the differentiation and mineralization of osteogenic cells by activating β‐catenin expression and the Wnt signaling pathway.^[^ ^18a^ ^]^ It is obvious that the Tai Chi pattern with a 2:1 ratio of MSCs to macrophages in the scaffolds had a more positive effect on the expression of intracellular osteo‐immunomodulation‐related genes and cytokines. These multicellular patterning scaffolds have the ability to improve osteogenesis by coordinating numerous signaling pathways between MSCs and macrophages, indicating the mutual regulatory effects of MSCs and macrophages in the multicellular patterns.

### 2.4 Effects of Multicellular Patterns with Different Cell Ratios on MSC‐Macrophage Cross‐Talk

**Figure 5:**
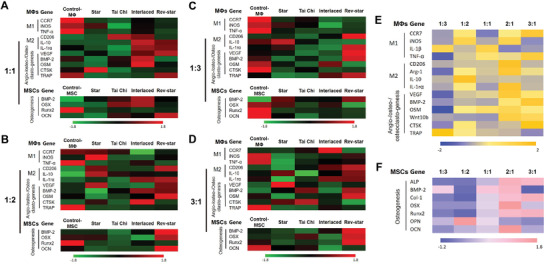
The modulatory effects of multicellular patterns with different cell ratios on MSC‐macrophage cross‐talk. A–D) when the ratio of MSCs to macrophages was A) 1:1, B) 1:2, C) 1:3, and D) 3:1, respectively, gene expression heatmap of immunomodulatory, angiogenic, osteogenic, and osteoclastic factors of the two kinds of cells cultured in different multicellular patterning scaffolds for 3 days via qRT‐PCR analysis. E) Gene expression heatmap of macrophages in the Tai Chi pattern with different cell ratios on day 3. F) Heatmap of osteogenic gene expression of MSCs in the Tai Chi pattern with different cell ratios on day 3. *n* = 4 for each scaffold type. Statistical interpretation was discussed in the Experimental Section. The different spatial distributions and cell ratios of MSCs and macrophages could trigger distinct “cross‐talk” between the two kinds of cells, thus affecting their differentiation behaviors and immunomodulatory functions.

Given the significant influence of patterned MSC‐macrophage spatial distribution on their intercellular communication, we continued to explore the modulatory effects of multicellular patterns with different cell ratios (1:1, 1:2, 1:3, and 3:1) on the gene expression of MSCs and macrophages. According to the gene expression heatmap in **Figure** **5**A–D, when the cell ratio of MSCs to macrophages was 1:1, macrophages from the Interlaced group expressed much higher levels of the M2 macrophage‐related genes (CD206, IL‐10, and IL‐1rα) and angiogenesis/osteogenesis‐related regulatory factor genes (VEGF, BMP‐2, and OSM) as well as lower levels of osteoclastic‐related genes (CTSK and TRAP) as compared with the other multicellular pattern groups. Moreover, MSCs in the Interlaced group showed a substantially greater increase in the expression of osteogenic marker genes (BMP‐2 and OSX) than those detected in other groups. This indicated that Interlaced pattern with a 1:1 ratio of MSCs to macrophages in the scaffold induced macrophage polarization toward a more anti‐inflammatory and prorepairing extreme phenotype, while also enhancing the osteogenic effect of MSCs. Interestingly, it was observed that when the cell ratio of MSCs to macrophages was 1:2, the Rev‐star pattern could substantially inhibit the inflammatory response of macrophages and promote the osteogenic differentiation of MSCs compared with the other patterns, with enhanced gene expression of anti‐inflammatory cytokine genes (CD206 and IL‐1rα) and osteogenesis‐related genes (VEGF, BMP‐2, OSM, OSX, and OCN) and reduced expression of proinflammatory genes (CCR7 and TNF‐*α*) and osteoclastic‐related genes (CTSK and TRAP). At a 1:3 ratio of MSCs to macrophages, the general observation was that, although macrophages within the Rev‐star pattern seemed to be polarized toward a more prohealing extreme phenotype based on the gene expression of M1 and M2 macrophage markers compared with the unpattern Control‐MΦ group, the expression of the most osteogenic genes in MSCs was downregulated. However, when the MSC: macrophage ratio was 3:1, the Rev‐star‐patterned coculture scaffolds performed better in terms of CD206, IL‐10, IL‐1rα, BMP‐2, OSM, and CTSK expression in macrophages and BMP‐2, OSX and Runx2 expression in MSCs, demonstrating a more excellent performance in inducing M2 macrophage activation and MSCs osteogenic differentiation. In addition, by comparing the effects of the Tai Chi pattern with different cell ratios on MSCs and macrophages, a more pronounced trend of driving macrophages toward anti‐inflammatory M2 phenotypic polarization was observed at a 2:1 ratio of MSCs to macrophages, accompanied by a significant increase in osteogenic and angiogenic factor expression as well as a decrease in osteoclastic factor expression (Figure 5E). Furthermore, the osteo‐immune microenvironment created by MSCs cocultured with macrophages at a cell ratio of 2:1 in the Tai Chi pattern was more conducive to osteogenic differentiation of MSCs than those created by MSCs to macrophages at 1:1, 1:2, 1:3, and 3:1 ratios, with widespread high expression of numerous osteogenic genes (Figure 5F). The original quantification data of the gene expression were presented in Figures S3–S7 (Supporting Information). Taken together, the results suggested that altering the spatial distribution and cell ratio of MSCs and macrophages in the scaffold triggered different “cross‐talk” between the two kinds of cells, which had a significant impact on their differentiation behaviors.

### 2.5 In Vivo Immunomodulatory Activities of Multicellular Patterning Bioactive Scaffolds

**Figure 6:**
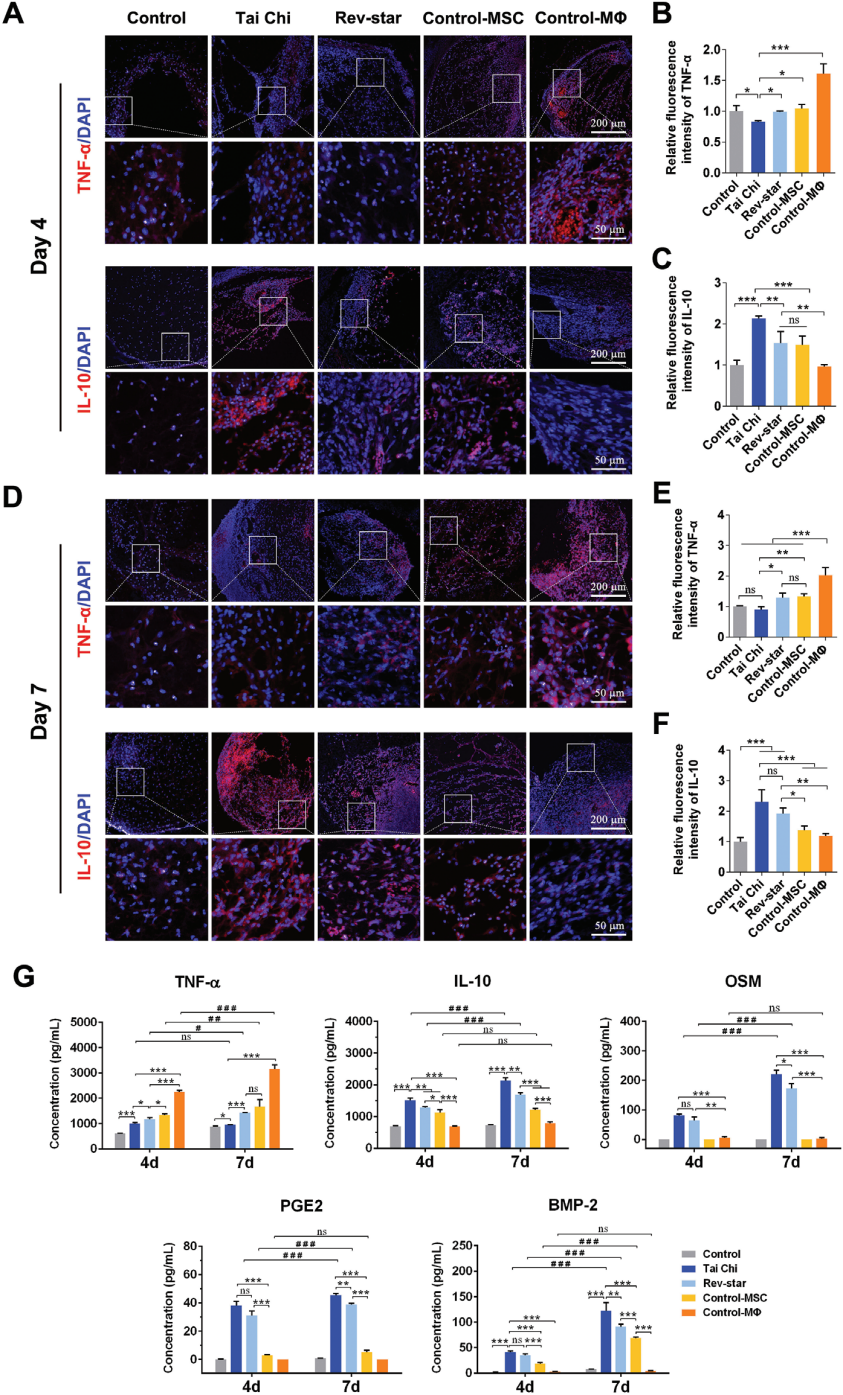
In vivo inflammatory response of multicellular patterning bioactive scaffolds after 4 and 7 days of subcutaneous implantation. A–F) Immunofluorescence staining and quantitative analysis of TNF‐α (proinflammatory factor) and IL‐10 (anti‐inflammatory factor) expression in different implant samples. Data presented as mean ± SD, *n* = 4 for each scaffold type, *p*‐values are calculated using one‐way ANOVA with Tukey correction, *p < 0.05, **p < 0.01, and ***p < 0.001; ns, not statistically significant. G) ELISA analyses of the secretion levels of pro‐inflammatory cytokine TNF‐α, anti‐inflammatory cytokines IL‐10, and PGE2, and osteo‐inductive factors OSM and BMP‐2 in tissues surrounding the scaffolds after 4 and 7 days of implantation. Error bars represent mean ± SD, *n* = 3 for each scaffold type, *p*‐values are calculated using one‐way ANOVA with Tukey correction, *p < 0.05, **p < 0.01, and ***p < 0.001; ns, not statistically significant. The multicellular coculture scaffolds with the Tai Chi pattern promoted the secretion of anti‐inflammatory factors and osteogenic factors while inhibiting the production of proinflammatory cytokines, exhibiting an enhanced in vivo osteo‐immunomodulatory effect.

Encouraged by the in vitro osteo‐immunomodulatory effect of multicellular patterning scaffolds, we further verified the in vivo interaction of MSCs and macrophages in multicellular patterning bioceramic scaffolds using a nude mouse subcutaneous implant model. Herein, the Tai Chi pattern scaffolds with a 2:1 ratio of MSCs to macrophages were chosen for implantation because the expression of osteogenic genes was demonstrated to be the highest, and the corresponding Rev‐star pattern scaffolds were used for comparison. After 4–7 days of implantation, the scaffolds were retrieved for immunofluorescence histochemical staining of TNF‐α (proinflammatory marker) and IL‐10 (anti‐inflammatory marker). The results showed a remarkably higher TNF‐α‐positive level and a significantly lower IL‐10‐positive level in the Control and Control‐MΦ groups than in the Tai Chi, Rev‐star, and Control‐MSC groups on day 4. The Tai Chi group had the lowest TNF‐α but highest IL‐10 expression of any groups (**Figure** **6**A). The Tai Chi and Rev‐star groups demonstrated a clear similarity in terms of decreased TNF‐α but increased IL‐10 expression from day 4 to day 7, as validated by quantitative analysis of fluorescence intensity (Figure 6B,C,E,F). It was evident that all multicellular pattern groups (Tai Chi and Rev‐star) containing MSC‐macrophage co‐culture expressed significantly more IL‐10 than unpatterned mono‐culture groups (Control‐MSC and Control‐MΦ) and the cell‐free loading group (Control) on day 7, with the Tai Chi pattern group expressing slightly higher than the Rev‐star pattern group and its TNF‐α‐positive level being the lowest relative to the other groups (Figure 6D).

Furthermore, the secretion levels of inflammatory cytokines in tissues surrounding the scaffold implants at 4 and 7 days postimplantation were detected by ELISA. The results are shown in Figure 6G. As expected, endogenous tissue cells and exogenous MSCs/macrophages grown in scaffolds with a Tai Chi‐shaped multicellular pattern secreted much less proinflammatory cytokine TNF‐α but more anti‐inflammatory (IL‐10 and prostaglandin E2 (PGE2)) and osteo‐inductive factors (OSM and BMP‐2) than scaffolds with a Rev‐star‐shaped multicellular pattern, which coincided with the transition of M1‐to‐M2 macrophages polarization. Moreover, we found that the concentrations of these anti‐inflammatory cytokines, as well as osteogenesis‐associated cytokines, increased continuously over time in the tissue sample around the scaffolds with MSC‐macrophage coculture patterns, illustrating good concordance with the immunostaining results mentioned previously. Indeed, in this multicellular patterning coculture environment, the interaction of MSCs with macrophages was complex. Their communication might form a feedback loop by means of juxtacrine or/and paracrine soluble mediators, which affected not only the immune properties of macrophages but also the differentiation potential of MSCs. In the light of current studies, OSM, PGE2, and BMP‐2 have arisen as the best recognized paracrine mediators of MSC‐macrophage cross‐talk. These cytokines may act synergistically to participate in the immunomodulatory osteogenesis process. OSM, in particular, is one of the key cytokines produced by macrophages that promotes bone formation in the early stages of fracture healing.^[^ ^21^ ^]^ According to recent studies, MSCs exhibit immunomodulatory activity in addition to their multidirectional differentiation potential and are capable of polarizing into two distinct immunophenotypic subtypes, namely MSC1 and MSC2.^[^ ^17^, ^22^ ^]^ MSC1 has a proinflammatory profile that favors the immune response during the initial stages of injury; on the contrary, MSC2 possesses anti‐inflammatory and immunosuppressive properties that aid in the repair of damaged tissues. However, MSCs are not spontaneously immunosuppressive.^[^ ^23^ ^]^ MSC‐mediated immunosuppression must be initiated by inflammatory cytokines such as interferon‐γ (IFN‐γ) and TNF‐α.^[^ ^24^ ^]^ Although these proinflammatory substances are generally thought to be the initiators of macrophage polarization toward the M1 extreme phenotype, when cocultured with MSCs, they in turn stimulate the production of immunomodulatory factors, such as PGE2, tumor necrosis factor‐stimulated gene‐6 (TSG6), and cyclooxygenase 2 (COX2) in MSCs.^[^ ^25^ ^]^ PGE2, as one of the major regulatory factors associated with the immunomodulatory function of MSCs, has been shown to increase IL‐10 secretion while inhibiting the production of pro‐inflammatory cytokines such as TNF‐α and interleukin‐12 (IL‐12), thereby modulating macrophage polarization and decreasing inflammation.^[^ ^26^ ^]^ As with the results of elevated PGE2 and IL‐10 levels detected by Nicolaidou et al. and most researchers in cocultures of inflammatory macrophages and MSCs,^[^ ^19^, ^26^, ^27^ ^]^ multicellular coculture patterns in our study, particularly the Tai Chi pattern, increased PGE2 and IL‐10 production while suppressing TNF‐α secretion, demonstrating strong modulatory effects on the immunomodulatory activity of MSCs and the inflammatory response of macrophages.

**Figure 7:**
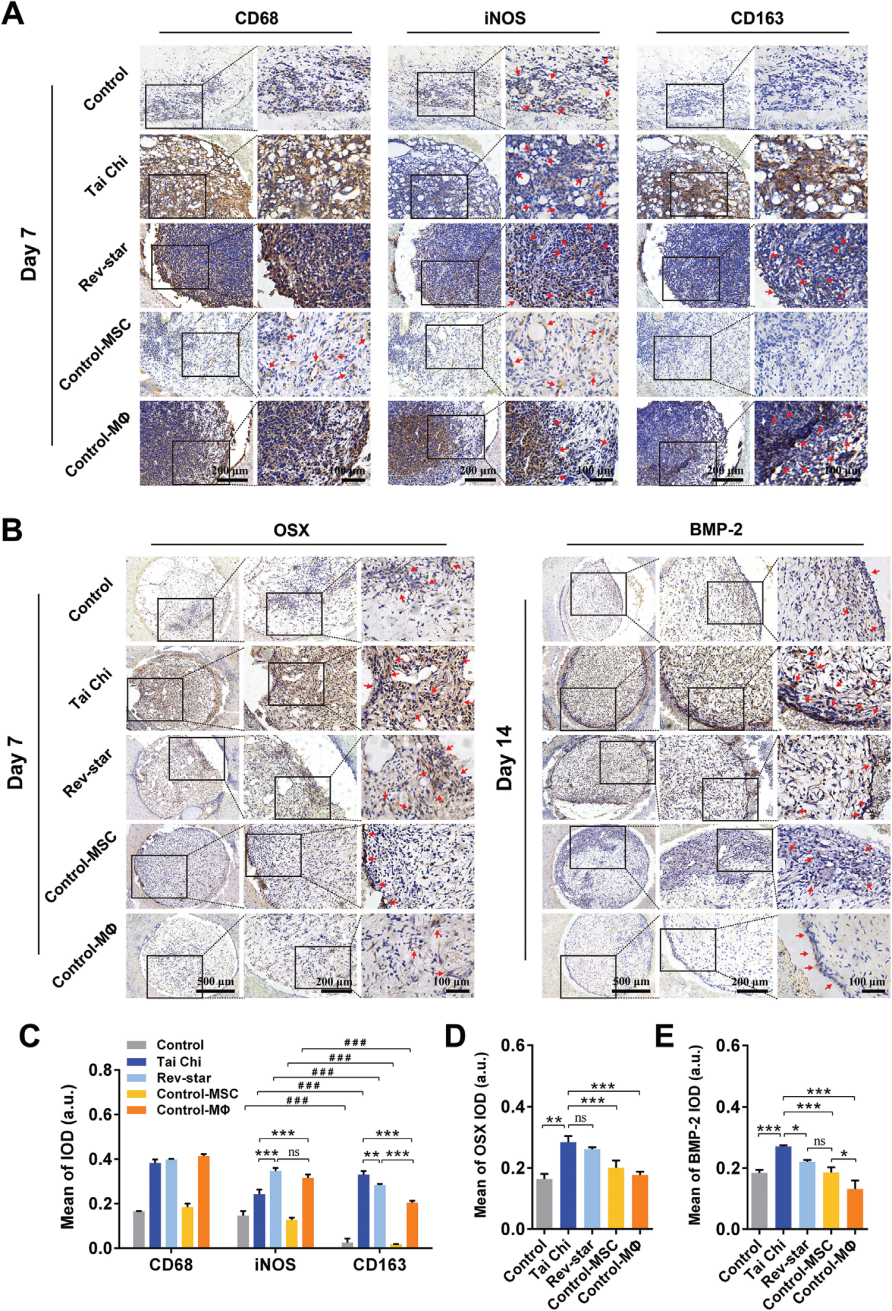
Evaluation of macrophage polarization and MSCs differentiation in the multicellular patterning bioactive scaffolds after 7 and 14 days of subcutaneous implantation. A,B) Representative immunohistochemical staining of CD68 (pan‐macrophage marker), iNOS (M1 macrophage surface marker), CD163 (M2 macrophage surface marker), OSX and BMP‐2 (osteogenic cytokines) in cell‐free group, MSCs or macrophages monoculture groups, Tai Chi‐patterned and Rev‐star‐patterned multicellular coculture groups with a 2:1 ratio of MSCs to macrophages after subcutaneous implantation for 7 and 14 days. The red arrows represent positively stained cells. C–E) Quantitative analysis of the mean integral optical density of C) CD68, iNOS, CD163, D) OSX, and E) BMP‐2 expressions. Data presented as mean ± SD, *n* = 3 for each scaffold type, *p*‐values are calculated using one‐way ANOVA with Tukey correction, *p < 0.05, **p < 0.01, and ***p < 0.001; ns, not statistically significant. The Tai Chi pattern in scaffolds activated macrophage polarization toward a more pro‐healing extreme M2 phenotype and significantly promoted the osteogenic differentiation of MSCs, providing a favorable osteo‐immune microenvironment for tissue regeneration.

Macrophage subpopulations in different samples were histologically immuno‐phenotyped using CD68 (a ubiquitous macrophage‘s membrane protein marker), iNOS (a specific M1 macrophage marker), and CD163 (a specific M2 macrophage marker) on days 7 and 14 postimplantation. The immunohistochemistry staining images and the semiquantitative results are shown in **Figure** **7**A,C. In the Tai Chi pattern group, more cells expressing CD68 and CD163 (CD68^+^ CD163^+^) were detected than CD68‐and iNOS‐positive cells (CD68^+^ iNOS^+^), and the activated state was maintained for 7–14 days. Whereas in the other groups, iNOS‐staining highly positive cells were more predominant than CD163‐positive cells. However, after 14 days of implantation, the expression of M2 macrophage marker CD163 was gradually increased in the Rev‐star pattern group and the Control‐MSC group, while the M1 macrophage marker iNOS was downregulated (Figure S8, Supporting Information). Instead, the main positively stained cells in the Control‐MΦ and Control groups were iNOS^+^ M1 macrophages. Meanwhile, more OSX‐positive cells were detected in the Tai Chi group than in the other groups at 7 days postimplantation (Figure 7B,D). Similar results were obtained for BMP‐2 expression at 14 days and were verified by the corresponding quantitative analysis (Figure 7B,E). Consistent with in vitro studies, our data demonstrated that the Tai Chi pattern endowed the scaffolds with enhanced immunomodulatory activity, promoting the secretion of immunomodulatory and osteogenic factors and inducing macrophage M2 polarization, which may help to alleviate the inflammation status caused by the scaffolds and construct a favorable microenvironment for tissue regeneration.

### 2.6 In Vivo Bone Regenerative Ability of Multicellular Patterning Bioactive Scaffolds

**Figure 8:**
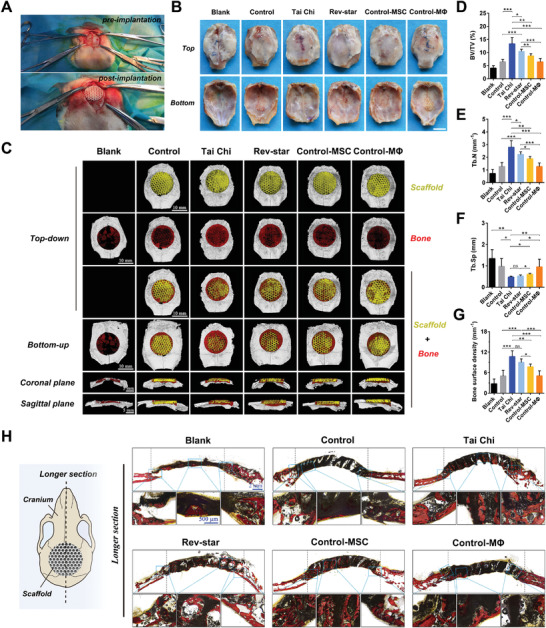
In vivo bone regeneration of multicellular patterning bioactive scaffolds after implanted in the cranial defect of rabbit models. A) Intraoperative digital photographs of the implantation of multicellular patterning scaffolds in rabbit cranial defects. B) Digital photographs and C) 3D reconstruction Micro‐CT images showing regenerated new bone in the defects without treatment and those treated with the cell‐free scaffolds, MSCs or macrophages monoculture scaffolds, Tai Chi‐patterned, and Rev‐star‐patterned multicellular coculture scaffolds with a 2:1 ratio of MSCs to macrophages after 12 weeks of postsurgery (red represents newly formed bone and yellow represents scaffolds). Scale bars, 10 mm. D–G) Quantitative analysis of Micro‐CT parameters in different treatment groups: D) BV/TV, E) Tb.N, F) Tb.Sp, and G) bone surface density. Data presented as mean ± SD, *n* = 6 for each scaffold type, *p*‐values are calculated using one‐way ANOVA with Tukey correction, *p < 0.05, **p < 0.01, and ***p < 0.001; ns, not statistically significant. H) Histological analysis of different scaffolds by Van Gieson's picrofuchsin staining after implantation for 12 weeks. In the middle of the samples, tissue sections with a longer length perpendicular to the implant were prepared and then used for histological staining analysis. Red color indicates bone tissue. Both the peripheral and internal scaffold channels of the Tai Chi group were filled with more newly formed bone tissue as compared with the control and Interlaced groups, indicating the significantly improved bone‐repair effect of multicellular coculture scaffolds with the Tai Chi pattern.

Multicellular coculture scaffolds with the Tai Chi pattern and Rev‐star pattern were implanted into rabbit cranial defects to evaluate the influence of different patterned spatial distribution between MSCs and macrophages on the osteogenic performance of bioceramic scaffolds (**Figure** **8**A). The implantation of MSCs or macrophages monoculture scaffolds (Control‐MSC and Control‐MΦ) and cell‐free scaffolds (Control) as well as cranial defects without treatment (Blank) were used as controls. The rabbits were sacrificed at 12 weeks postsurgery to assess the bone‐repair effect of the multicellular patterning bioactive scaffolds. None of the samples showed inflammation (Figure 8B). The 3D reconstructed microcomputed tomography (Micro‐CT) images showed the newly formed bone (red) within scaffolds (yellow) on the transverse top and bottom surfaces as well as coronal and sagittal longitudinal sections (Figure 8C). It could be seen that more new bone tissue was formed in the Tai Chi group than in the other five groups. These bone tissues grew into the channels of the Tai Chi‐patterned coculture scaffolds. Furthermore, statistical analysis demonstrated that the bone volume/total volume (BV/TV) value, trabecular number (Tb.N), and bone surface density of the multicellular pattern groups (Tai Chi and Rev‐star) were obviously higher than those of the unpatterned control groups (Control‐MSC, Control‐MΦ, Control, and Blank), with the Tai Chi group exhibiting the highest values. (Figure 8D–G). Opposite trends were obtained when comparing the trabecular separation (Tb.Sp) values, suggesting the excellent osteogenic ability of Tai Chi‐patterned coculture scaffolds.

Histological analysis of Van Gieson's picrofuchsin staining further demonstrated that peripheral host bone grew closely to the scaffolds in all groups. However, the new bone area appeared to be greater in the Tai Chi and Rev‐star groups than in the other groups. From the staining images of longitudinal sections with different lengths (Figure 8H; and Figure S9, Supporting Information), it is observed that most of the channels in both the periphery and interior of the two multicellular patterning scaffolds were filled with newly formed bone tissue (red). In contrast, new bone formation in the unpatterned monoculture groups was mainly detected on the periphery of the scaffolds, and the new bone volume was even limited in the cell‐free scaffolds and blank groups, where most of the channels in the central region of the scaffolds remained vacant. Therefore, the above results demonstrated that the patterned spatial distribution of MSCs and macrophages had a significant regulatory effect on the immunomodulatory osteogenesis of scaffolds in vivo. Tai Chi‐patterned coculture scaffolds with a 2:1 ratio of MSCs to macrophages effectively promoted bone regeneration and repair.

## 3 Conclusion

In summary, a multicellular patterning scaffold was successfully prepared by loading MSCs and macrophages into the corresponding channels of honeycomb‐like 3D‐printed bioceramic scaffolds. It broke through the limitation of only being able to observe 2D “cross‐talk” between MSCs and macrophages mediated by bioceramic scaffolds, and enabled the patterned spatial distribution and spatial interactions of multiple tissue cells within the 3D structure. Moreover, it was found that different structured spatial distributions and cell ratios of MSCs and macrophages in the scaffolds could trigger distinct intercellular “cross‐talk,” thereby stimulating their differentiation behaviors and scaffold‐mediated tissue regeneration. Our results showed that when the ratio of MSCs to macrophages was 2:1, the Tai Chi pattern could induce the macrophages to polarize toward a more anti‐inflammatory (M2) phenotype with an elevated secretion of the prohealing cytokines as compared with other patterns. Concomitantly, the resulting immune environment significantly promoted the osteogenic differentiation of MSCs and subsequent bone tissue regeneration in vivo. This may be associated with the activation of BMP‐Smad, OSM/OSMR, and Wnt/β‐catenin signaling pathways in MSCs mediated by macrophage‐derived paracrine signaling mediators (BMP‐2, OSM, and Wnt10b). Obviously, the Tai Chi pattern was able to endow the scaffolds with enhanced immunomodulatory function and bone‐forming ability. Despite this, the mechanism of multicellular synergistic effects involved in the process needs to be further identified through the interactions between the signaling pathways. Apart from that, it should be noted that the local immune response after biomaterial implantation may not be restricted to macrophage activation. Considering its clinical application, there are still some issues to be investigated. Future research will focus on other immune cells, such as neutrophils in the innate response and T cells in the adaptive response, and their contributions to multicellular patterning scaffold‐guided neo‐bone regeneration. All in all, this work proposes an engineering strategy for inducing high‐efficiency bone regeneration through the manipulation of cell distribution in a 3D microenvironment, which may provide insight for the design of tissue‐engineered multicellular scaffolds.

## 4 Experimental Section

### Preparation and Characterization of Honeycomb‐Like Bioceramic Scaffolds

Ca, Si, and P‐containing NAGEL (Ca_7_Si_2_P_2_O_16_) ceramic powder was synthesized by a sol–gel process according to the previous publication.^[^ ^28^ ^]^ 50 g of NAGEL bioceramic powder and 2.5 g of 45S5 bioactive glass powder were ball milled with 52.5 g of photosensitive resin to obtain the printable precursor slurry. Then, multichannel honeycomb‐like scaffolds with controllable pore orientation and pore size were fabricated by a DLP laser rapid prototyping 3D printer (AUTOCERA‐M, Beijing Ten Dimensions Technology Co. Ltd., China) following computer‐aided design models. After washing in ethanol via ultrasonic cleaning, the green bodies of the scaffolds were sintered at 1400 °C for 3 h to obtain the pure bioceramic scaffolds. The sintered NAGEL bioceramic scaffolds (diameter: 15 mm, height: 2.5 mm) were characterized using X‐ray diffraction (XRD, D8 ADVANCE, BRUKER AXS GMBH, Karlsruhe, Germany) and scanning electron microscopy (SEM, S‐4800, Hitachi, Japan).

### Patterned Loading of Multiple Cell Lines Within the Multichannel Structure of Bioceramic Scaffolds

The RAW 264.7 macrophage cell line (MΦ) was purchased from the cell bank of the Chinese Academy of Sciences and cultured in Dulbecco's modified Eagle's medium (DMEM) with high‐glucose (Gibco, USA) containing 1% penicillin‐streptomycin (Gibco, USA) and 10% fetal bovine serum (FBS) (Gibco, USA), with the culture medium changed every other day. The mouse bone marrow‐derived mesenchymal stem cells (MSCs) (Shanghai Zhong Qiao Xin Zhou Biotechnology Co. Ltd., Shanghai, China) were cultured in MSCM culture medium (Sciencell, America), with the medium replaced every 2 days. New Zealand white rabbit bone marrow‐derived MSCs were purchased from Cyagen Biotechnology Co., Ltd. (Cyagen, Guangzhou, China) and cultured in rabbit MSCs culture medium (Cyagen, Guangzhou, China). New Zealand white rabbit bone marrow‐derived primary macrophages were purchased from iCell Biotechnology Co., Ltd. (iCell, Shanghai, China) and cultured in macrophage conditioned medium (Cyagen, Guangzhou, China), which can be polarized toward different phenotypes in response to external stimuli and have a high capacity for polarization (Figure S1, Supporting Information). All cells were maintained at 37 °C in 100% humidified air containing 5% CO_2_ and cultured according to the instructions. Only the 4–7 passages of MSCs were used in this study.

Under sterile conditions, MSCs and macrophages were, respectively, seeded on the corresponding channels of bioceramic scaffolds with a cell amount of 5 × 10^5^ cells per scaffold (the cell ratios of MSCs to macrophages: 1:1, 1:2, 2:1, 1:3, and 3:1, respectively) according to four typical cell distribution patterns (star, Tai Chi, interlaced, and reverse star). In brief, 3 µL of macrophages suspension per channel was first added into the specific channel of the scaffold, and then placed in the incubator at 37 °C and 5% CO_2_. After incubation for 30 min, 2.5 µL of MSCs suspension per channel was seeded. Due to the extremely small amount of liquid, the solution quickly penetrated into the bioceramic scaffolds, and the cells could be intercepted on the surface of the inner walls of the channels. After incubating for another 1 h, the cell‐laden scaffolds were transferred to new 24‐well plates including DMEM/MSCM (1:1, v/v) mixed culture medium for further coculture. Scaffolds containing only MSCs or only macrophages and cell‐free loading scaffold were used as controls, denoted as Control‐MSC, Control‐MΦ, and Control, respectively. MSCs and RAW 264.7 macrophages grown in the scaffolds were digested and collected on days 2 and 5. Macrophages were then isolated from the mixed cells by using mouse CD11b MicroBeads (Miltenyi Biotec, Germany). Subsequently, the separated MSCs and RAW 264.7 cells were reincubated on new 6‐well plates. After 4 h, the viabilities of these two kinds of cells were assessed by CCK‐8 analysis, respectively (*n* = 4). Unless otherwise indicated, all multicellular patterning bioactive scaffolds were prepared using mouse bone marrow‐derived MSCs and RAW 264.7 macrophages.

### Multicellular Pattern Fluorescence Imaging and Cell Morphological Observation

To visualize the multicellular pattern in the scaffolds and the cell growth in the interior of channels, MSCs and RAW 264.7 macrophages were, respectively, stained with a red‐fluorescence probe (CellTracker TM Deep Red, Life Technologies Co. Ltd., USA) (CTDR) and a green‐fluorescence probe (CellTracker TM Green CMFDA, Life Technologies Co. Ltd., USA) (CTG) in advance of seeding cells. The nuclei of cells were stained with 4,6‐diamino‐2‐phenyl indole (DAPI; Sigma‐Aldrich, USA). After a 3‐ or 5‐day cultivation, confocal images of the 3D cell pattern and the cell growth in the interior of channels were obtained on a laser scanning confocal microscopy (CLSM, Leica TCS SP8, Leica Microsystems, Germany). The cell morphology on the scaffolds after culturing for 3 days was further observed by SEM after being fixed by 2.5% glutaraldehyde, dehydrated through gradient ethanol solutions (30%, 50%, 70%, 90%, 95%, and 100%), vacuum dried, and sprayed with gold.

### Gene Expression Analysis

qRT‐PCR was performed to investigate gene expression in MSCs and RAW 264.7 macrophages stimulated by different multicellular patterns. After culturing for 3 days, all cells were digested with 0.05% trypsin/ethylene diamine tetraacetic acid (EDTA) and collected from the scaffolds. RAW 264.7 macrophages were then isolated from the cocultures by using mouse CD11b MicroBeads. Subsequently, the total RNA of two kinds of cells was separately extracted with Trizol reagent (Invitrogen, USA) and then reverse‐transcribed to cDNA using PrimeScript Master Mix Kit (TaKaRa, Japan) following the manufacturer's instructions. qRT‐PCR reaction was carried out using gene‐specific primers and the TB Green Premix Ex Taq Kit (TaKaRa, Japan) on StepOnePlus Real‐Time PCR Systems (Applied Biosystems, USA). The fold increase or decrease was determined relative to the Control‐MSC or Control‐MΦ group after normalizing using the 2^−ΔΔCt^ method (*n* = 4). The primer sequences used in qRT‐PCR are listed in Table S1 (Supporting Information).

### Quantification of Released Growth Factors and Cytokines

On day 7, the supernatants of MSCs and macrophages cultured on scaffolds were collected at day 7. The content of pro‐inflammatory (TNF‐α, iNOS), anti‐inflammatory cytokines (IL‐10, Arg‐1), and osteogenic factors (OSM, BMP‐2) secreted by cells in the supernatant was detected with corresponding ELISA kits (Cusabio, China). All ELISAs were conducted according to the manufacturer's instructions.

### Immunofluorescence Staining

To detect macrophage polarization and the expression of specific osteogenesis‐related proteins in MSCs, the immunofluorescent protein images of these cells were observed by CLSM after culturing for 3 days. Briefly, cells were fixed with 2.5% glutaraldehyde solution for 15 min, permeabilized with 0.1% Triton X‐100 for 5 min, and nonspecific binding blocked with 5% BSA for 30 min. Subsequently, macrophages in different samples were incubated with primary antibody against CCR7 (catalog no. MAB3477, R&D Systems, USA) at a 1:60 dilution at 4 °C overnight. Then, the cells were further separately incubated with Alexa Fluor 647‐conjugated CD206 antibody (dilution 1:50; catalog no. 141 712, Biolegend, USA) and Alexa Fluor 488‐conjugated antirat secondary antibody (dilution 1:500; catalog no. ab150157, Abcam, USA) for 1 h, followed by 15 min of nuclear staining with DAPI. Similarly, MSCs were incubated with primary antibody against BMP‐2 (dilution 1:100; catalog no. ab214821, Abcam, USA) and Alexa Fluor 647‐conjugated antirabbit secondary antibody (dilution 1:500; catalog no. ab150079, Abcam, USA) following the same experimental procedure. After washing with phosphate buffer saline (PBS), the nuclei and cytoskeleton were counterstained with DAPI and FITC‐labeled phalloidin, and repeatedly washed before imaging using CLSM.

### ALP Staining and OCN Immunofluorescent Staining

The osteogenic properties of MSCs’ paracrine products were further evaluated by ALP and OCN staining. After incubating for 7 days, MSCs digested and isolated from the cocultures were stained with an ALP staining kit (Beyotime, China) after reseeding in 24‐well culture plates for 12 h. Primary antibody (dilution 1:100; catalog no. ab93876, Abcam, USA) and Alexa Fluor 594‐conjugated antirabbit antibodies (dilution 1:200; catalog no. ab150080, Abcam, USA) were used for immunofluorescence staining of OCN according to the procedure outlined in the preceding section.

### In Vivo Osteo‐Immunomodulation Assessment

The multicellular patterning bioactive scaffolds incubated in vitro for 1 day were subcutaneously implanted in the backs of 6–8 week old Balb/c nude mice, which were narcotized by pentobarbital (40 mg kg^−1^) in prior. After 4, 7, and 14 days of implantation, the mice were sacrificed to retrieve the samples for detecting the interaction of MSC‐macrophage patterned on bioceramic scaffolds in a 3D in vivo environment. Animals were randomly divided into five groups for implantation with the above‐mentioned two different multicellular patterning scaffolds (Tai Chi and Interlaced), only MSCs‐ or only macrophages‐laden scaffolds (Control‐MSC and Control‐MΦ) and cell‐free scaffolds (Control) (*n* = 6). All animal experiments were conducted in accordance with protocols approved by the Institutional Animal Care and Use Committee of Nanjing First Hospital, Nanjing Medical University (No. DW201902).

### In Vivo Osteo‐Immunomodulation Assessment—Immunofluorescence and Immunohistochemical Analyses of Tissue Sections

Retrieved implants were fixed in 4% paraformaldehyde for 24 h, decalcified in 10% EDTA (pH 7.4) for about 8 weeks. Then, the samples were embedded in paraffin and sectioned at a 7 µm thickness. Before immunoassay, antigen retrieval, endogenous peroxidase cancellation, and bovine serum albumin blocking were performed sequentially. Sections of samples from 4 and 7 days postimplantation were incubated overnight at 4 °C with the antibodies TNF‐α (dilution 1:100, catalog no. ab215188, Abcam, USA) and IL‐10 (dilution 1:50, catalog no. ab189392, Abcam, USA). Primary antibodies were then visualized with Alexa Fluor 647‐conjugated antirabbit secondary antibody. DAPI was used for nuclear staining. Fluorescent images of the sections were taken using CLSM. The Image‐Pro Plus 6.0 software (Media Cybernetics, USA) was used to obtain and quantitatively analyze the intensity of the immune signal. All specimens have been repeated in three parts. The sections for immunohistochemical staining were incubated with primary antibodies against CD68 (dilution 1:500, catalog no. ab125212, Abcam, USA) for pan‐macrophage marker, iNOS (dilution 1:100, catalog no. ab115819, Abcam, USA) for a M1 marker, CD163 (dilution 1:500, catalog no. ab182422, Abcam, USA) for a M2 maker, and OSX (dilution 1:100, catalog no. ab215188, Abcam, USA) and BMP‐2 for osteogenesis markers at appropriate dilution overnight at 4 °C. After rinsing thoroughly in PBS, the sections were incubated with a biotinylated anti‐rabbit secondary antibody and HRP‐labeled‐streptavidin (catalog no. ab64261, Abcam, USA), and then developed with Diaminobenzidine (DAB). Nuclei were revealed with hematoxylin staining. The positive expression cells in different samples were observed by optical microscopy. Quantitative image assessment of immunohistochemical staining was performed by Image‐Pro Plus 6.0 software.

### In Vivo Osteo‐Immunomodulation Assessment—Cytokine Measurements

The fresh harvested implants were ground with lysate (containing protease inhibitor) in liquid nitrogen, and subsequently centrifuged for 15 min at 12 000 g and 4 °C to obtain the supernatant of the tissue lysate, which was then stored at −80 °C before use. The secretion of inflammatory or osteogenic cytokines from tissues surrounding the scaffold implants, TNF‐α, IL‐10, PGE2, BMP‐2, and OSM, was examined with ELISA kits (Cusabio, China) following the manufacturer's instructions.

### In Vivo Osteogenesis Assessment

All mouse cell lines were replaced with New Zealand white rabbit‐derived cells for the preparation of multicellular patterning bioactive scaffolds. The rabbit cranial defect models were applied to investigate the immunomodulatory osteogenesis of these scaffolds. All New Zealand white rabbits were treated according to guidelines approved by the Institutional Animal Care and Use Committee of Nanjing First Hospital, Nanjing Medical University (No. DW201902). In brief, after being anesthetized, the calvaria of rabbits (2.5–3 kg) were exposed to a trephine drill under constant irrigation, and 15 mm diameter craniotomy defects were created in the middle of the parietal bone with care to avoid injury to the underlying dura mater. Immediately after the bone debris was removed, the cranial defect was rinsed with saline solution, and scaffolds incubated in vitro for 7 days were implanted, followed by suture. The rabbits were randomly divided into six groups (*n* = 6): Blank group (without any implants), Control group, Tai Chi group, Rev‐star group, Control‐MSC group, and Control‐MΦ group. After 12 weeks of implantation, all the rabbits were sacrificed, and their craniums were harvested and fixed in 10% formalin for further analysis.

### In Vivo Osteogenesis Assessment—Micro‐CT Analysis

To analyze neo‐bone formation, the harvested samples were scanned by a Skyscan 1174 micro‐CT system (Bruker, Germany) at a resolution of 13 µm pixels. Visualization and reconstruction of the data were obtained using the NRecon and CTvox imaging software. Bone volume density (bone volume/tissue volume, BV/TV [%]), bone surface density, trabecular number (Tb.N; mm^−1^), and trabecular separation (Tb.Sp; mm) were measured with CTAn software (Bruker Skyscan, Germany).

### In Vivo Osteogenesis Assessment—Histological Characteristics

For evaluation of bone histology, the fixed tissues were dehydrated and embedded in methyl methacrylate. After that, in the middle of the samples and near the periphery, 500 µm thick sections of different lengths perpendicular to the implants were prepared using a microtome with a diamond blade (Leica Microsystems SP 1600, Nussloch, Germany). After being grinded and polished, standard Van Gieson's picrofuchsin staining was performed, and the tissue sections were examined by optical microscopy.

### Statistical Analysis

GraphPad Prism 8.0 software (GraphPad Software Inc., San Diego, CA) was used for the statistical data analysis. All data were examined in at least three independent experiments unless otherwise stated. Data were presented as mean ± standard deviation (SD) with sample size *n* ≥ 3. Multiple comparisons were assessed using one‐way analysis of variance (ANOVA). The analysis of variances followed by Tukey's post‐hoc test was employed in this work, and the *p* values for statistical significance are represented with stars (* *p* < 0.05, ** *p* < 0.01, *** *p* < 0.001).

## Conflict of Interest

The authors declare no conflict of interest.

## Author Contributions

B.Z. and C.W. conceived and initiated the project and wrote the manuscript. B.Z. and Y.S. designed and performed experiments. B.Z., F.H., Y.W., M.Z., C.Q., and H.Z. performed animal surgeries. B.Z. and X.Y. collected and analyzed all the data. C.W. supervised the project.

## Supporting information

Supporting Information

## Data Availability

The data that support the findings of this study are available from the corresponding author upon reasonable request.
