# Supplementary material for: Cells‐Micropatterning Biomaterials for Immune Activation and Bone Regeneration
Source: Adv Sci (Weinh). 2022 Apr 28;9(18):2200670. doi: 10.1002/advs.202200670 (PMC9218778; doi:10.1002/advs.202200670)
Supplement: Supplementary file 1 — Supporting Information [file ADVS-9-2200670-s001.pdf]

ADVANCED SCIENCE
Open Access

Supporting Information

for *Adv. Sci.*, DOI 10.1002/advs.202200670

Cells-Micropatterning Biomaterials for Immune Activation and Bone Regeneration

*Bingjun Zhang, Fei Han, Yufeng Wang, Yuhua Sun, Meng Zhang, Xiaopeng Yu, Chen Qin, Hongjian Zhang and Chengtie Wu**

Supporting Information

Cells-micropatterning Biomaterials for Immune Activation and Bone Regeneration

*Bingjun Zhang, Fei Han, Yufeng Wang, Yuhua Sun, Meng Zhang, Xiaopeng Yu, Chen Qin, Hongjian Zhang, Chengtie Wu**

Dr. B. Zhang, Dr. F. Han, Mr. Y. Wang, Dr. Y. Sun, Mr. M. Zhang, Mr. X. Yu, Ms. C. Qin, Mr. H. Zhang, Prof. C. Wu
State Key Laboratory of High Performance Ceramics and Superfine Microstructure, Shanghai Institute of Ceramics, Chinese Academy of Sciences, Shanghai 200050, P. R. China

Mr. M. Zhang, Mr. X. Yu, Ms. C. Qin, Mr. H. Zhang, Prof. C. Wu
Center of Materials Science and Optoelectronics Engineering, University of Chinese Academy of Sciences, Beijing 100049, P. R. China

* Corresponding author: Chengtie Wu

E-mail: chengtiewu@mail.sic.ac.cn (C. Wu)

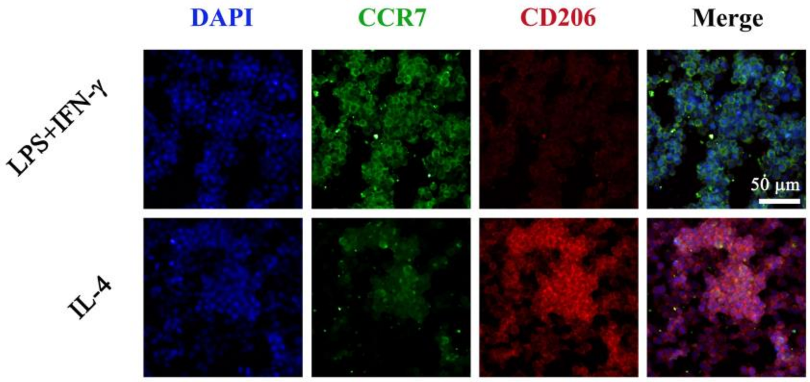

Figure S1. CCR7 (M1 polarized marker, green) and CD206 (M2 polarized marker, red) immunofluorescent staining images of rabbit bone marrow-derived primary macrophages. Primary macrophages treated with LPS+IFN-γ had stronger CCR7 expression, indicating that the cells were polarized to the M1 phenotype, while more remarkable expression of the CD206 signal could be observed in cells treated with IL-4, suggesting that the cells were stimulated toward M2-like phenotype-polarization. These macrophages were capable of polarizing toward different phenotypes in response to external stimuli, showing a good potential for polarization.

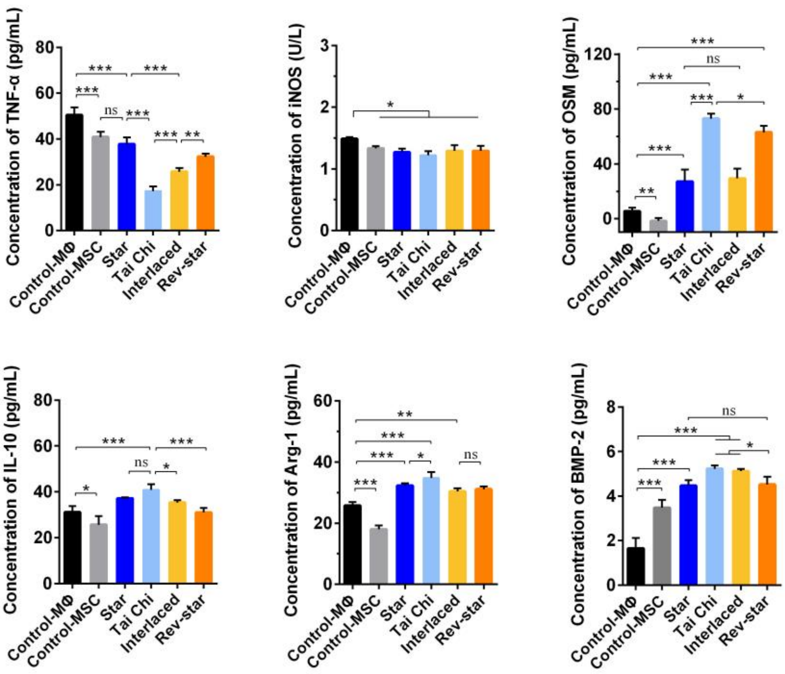

Figure S2. The secretion levels of inflammatory and osteogenic factors in cells cultured on scaffolds for 7 days. Compared with other patterns, the Tai Chi pattern significantly promoted the secretion of anti-inflammatory factors (IL-10, Arg-1) and osteogenic factors (OSM, BMP-2) while inhibiting the production of pro-inflammatory cytokines (TNF-α, iNOS), exhibiting an enhanced osteo-immunomodulatory effect. Data presented as mean ± SD, n = 3 for each scaffold type, p-values are calculated using one-way ANOVA with Tukey correction, $*p < 0.05$, $**p < 0.01$, and $***p < 0.001$; ns, not statistically significant.

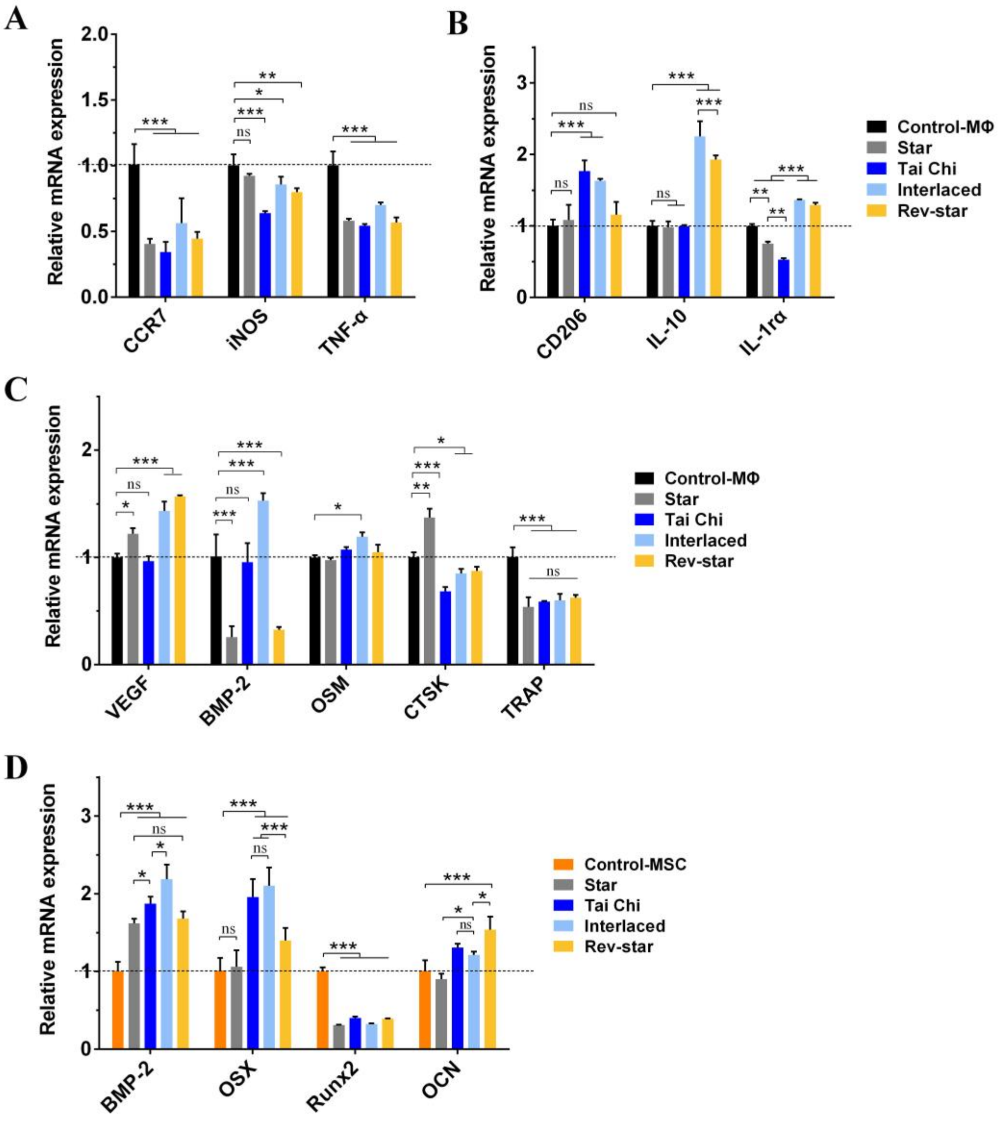

Figure S3. Relative gene expressions of macrophages and MSCs in different multicellular patterning bioactive scaffolds with a 1:1 ratio of MSCs to macrophages. (A-B) Gene expression of surface markers and pro/anti-inflammatory cytokines of macrophages in different scaffolds on day 3. (C) Gene expression of angiogenic, osteogenic, and osteoclastic factors of macrophages on day 3. (D) Expression of osteogenesis-related genes of MSCs in different scaffolds on day 3. Data presented as mean ± SD, n = 4 for each scaffold type, *p*-values are calculated using one-way ANOVA with Tukey correction, $*p < 0.05$, $**p < 0.01$, and $***p < 0.001$; ns, not statistically significant. Compared with other multicellular co-culture patterns, MSCs in the Interlaced pattern stimulated the polarization of macrophages toward a more anti-inflammatory extreme phenotype, and simultaneously macrophages also significantly enhanced the osteogenic

effect of MSCs.

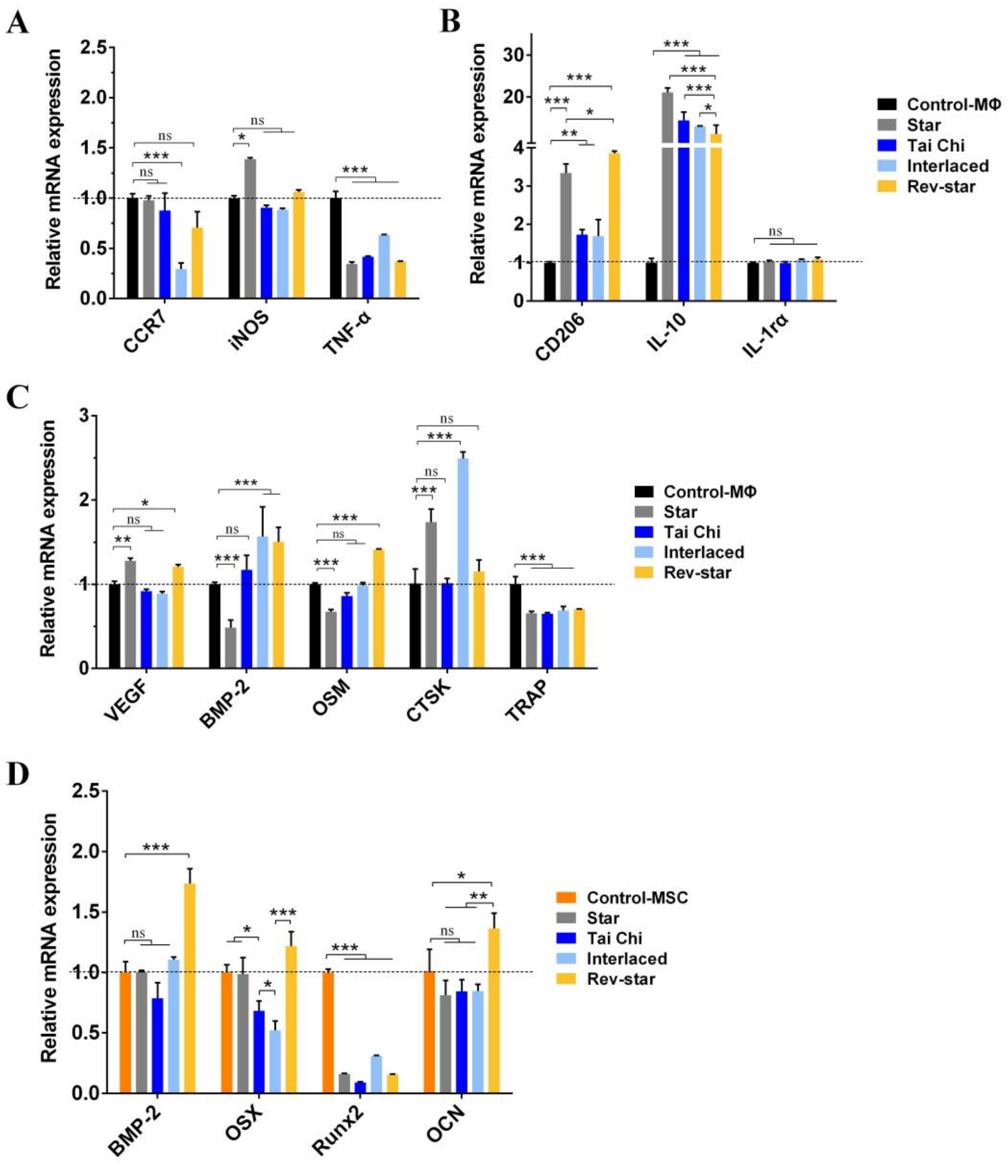

Figure S4. Relative gene expressions of macrophages and MSCs in different multicellular patterning bioactive scaffolds with a 1:2 ratio of MSCs to macrophages. (A-B) Gene expression of surface markers and pro/anti-inflammatory cytokines of macrophages in different scaffolds on day 3. (C) Gene expression of angiogenic, osteogenic, and osteoclastic factors of macrophages on day 3. (D) Expression of osteogenesis-related genes of MSCs in different scaffolds on day 3. Data presented as mean ± SD, n = 4 for each scaffold type, *p*-values are calculated using one-way ANOVA with Tukey correction, **p* < 0.05, ***p* < 0.01, and ****p* < 0.001; ns, not statistically significant. The Rev-star pattern significantly inhibited macrophage inflammatory response and promoted osteogenic differentiation of MSCs as compared with other

multicellular patterns.

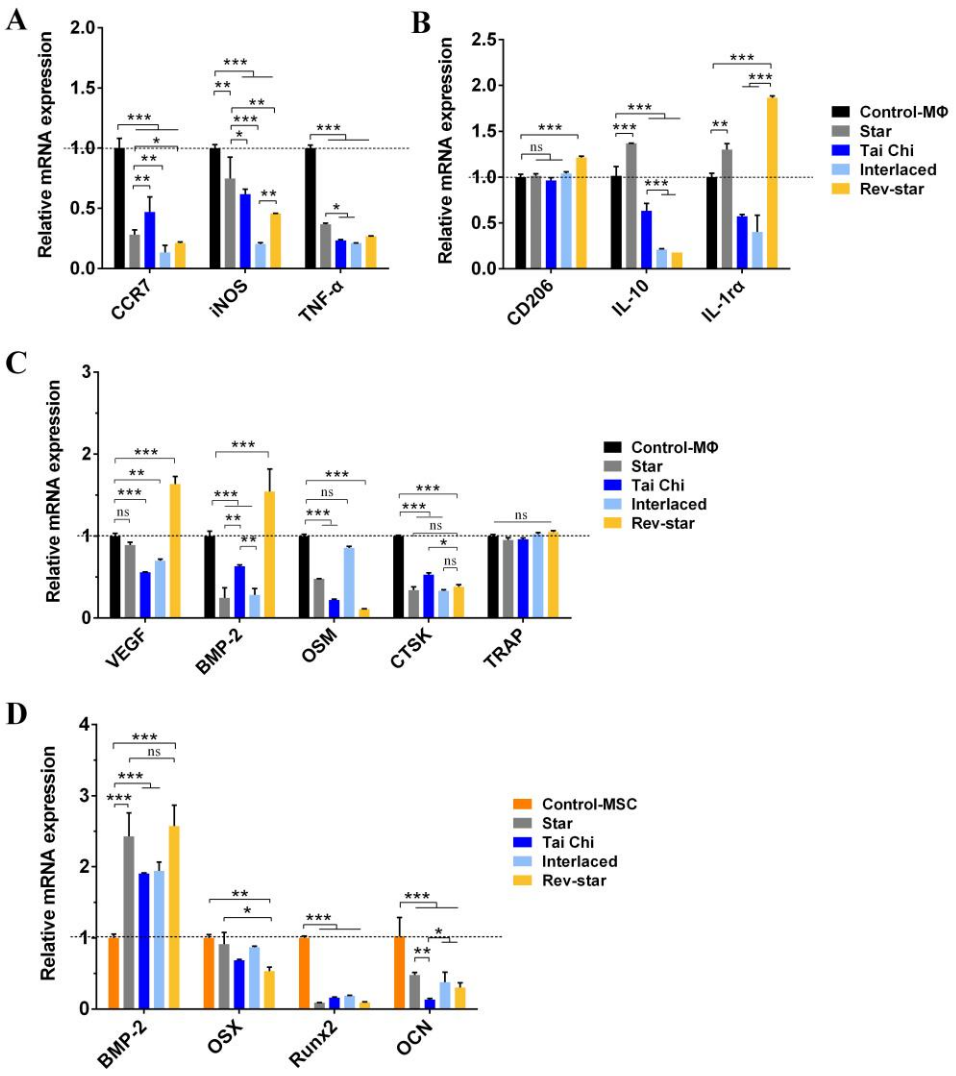

Figure S5. Relative gene expressions of macrophages and MSCs in different multicellular patterning bioactive scaffolds with a 1:3 ratio of MSCs to macrophages. (A-B) Gene expression of surface markers and pro/anti-inflammatory cytokines of macrophages in different scaffolds on day 3. (C) Gene expression of angiogenic, osteogenic, and osteoclastic factors of macrophages on day 3. (D) Expression of osteogenesis-related genes of MSCs in different scaffolds on day 3. Data presented as mean ± SD, n = 4 for each scaffold type, *p*-values are calculated using one-way ANOVA with Tukey correction, *$p < 0.05$, **$p < 0.01$, and ***$p < 0.001$; ns, not statistically significant. Although macrophages in the Rev-star pattern seemed to be polarized toward a more pro-healing extreme phenotype based on the gene expression of M1 and M2 macrophage markers compared with the unpattern Control-MΦ group, the expression of the

most osteogenic genes in MSCs was downregulated.

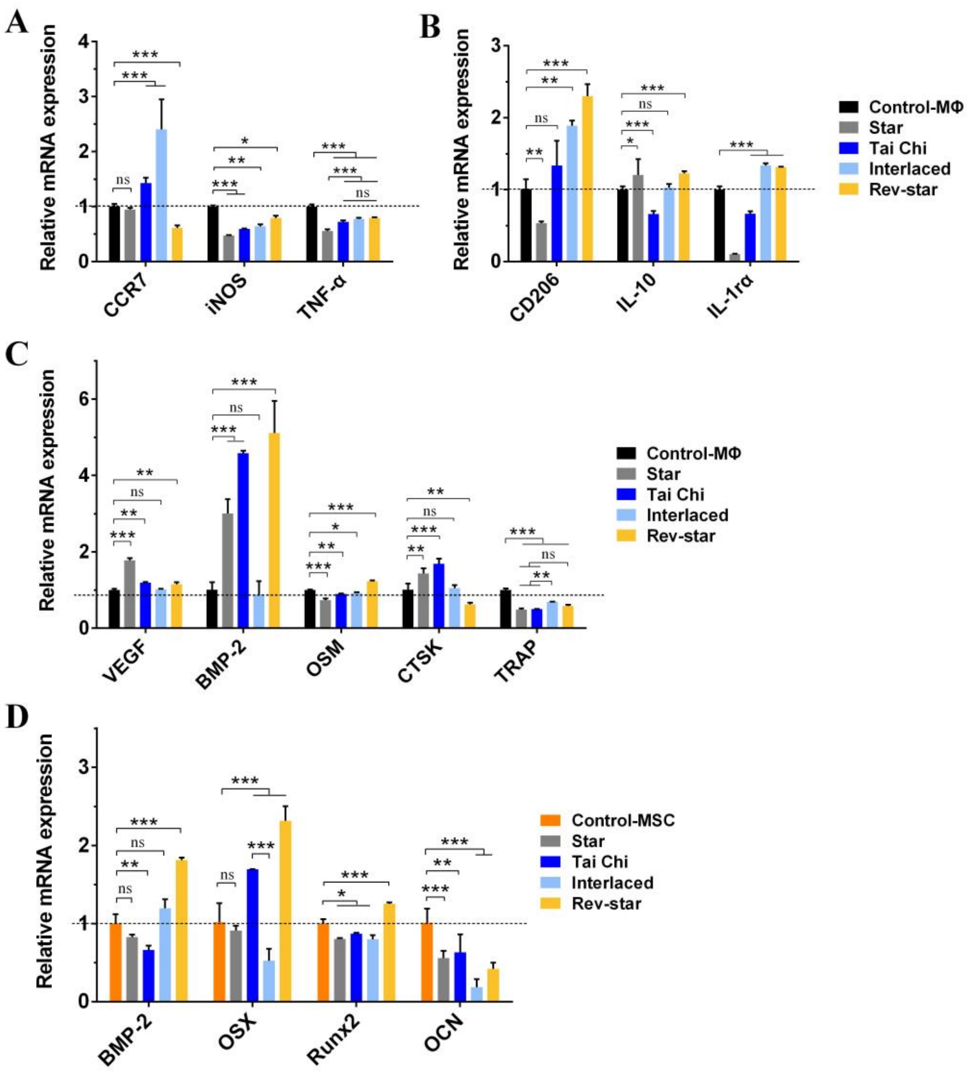

Figure S6. Relative gene expressions of macrophages and MSCs in different multicellular patterning bioactive scaffolds with a 3:1 ratio of MSCs to macrophages. (A-B) Gene expression of surface markers and pro/anti-inflammatory cytokines of macrophages in different scaffolds on day 3. (C) Gene expression of angiogenic, osteogenic, and osteoclastic factors of macrophages on day 3. (D) Expression of osteogenesis-related genes of MSCs in different scaffolds on day 3. Data presented as mean ± SD, n = 4 for each scaffold type, *p*-values are calculated using one-way ANOVA with Tukey correction, *$p < 0.05$, **$p < 0.01$, and ***$p < 0.001$; ns, not statistically significant. When the cell ratio of MSCs to macrophages was 3:1, the Rev-star pattern performed better in stimulating macrophages toward M2-like phenotype-polarization and promoting osteogenic differentiation of MSCs.

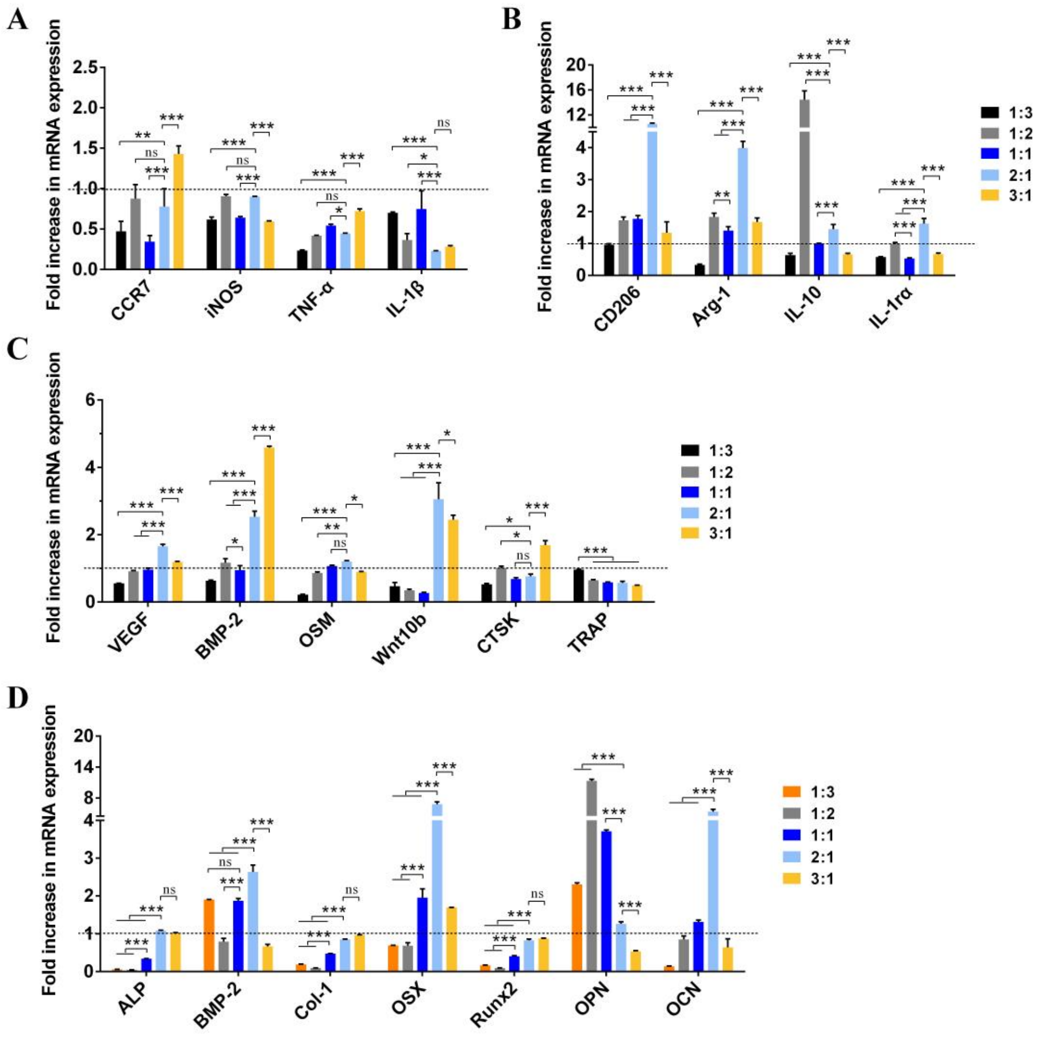

Figure S7. Relative fold in gene expression of macrophages and MSCs in the Tai Chi pattern with different cell ratios. (A-B) Relative fold in gene expression of surface markers and pro/anti-inflammatory cytokines of macrophages in different scaffolds on day 3. (C) Relative fold in gene expression of angiogenic, osteogenic, and osteoclastic factors of macrophages on day 3. (D) Relative fold in expression of osteogenesis-related genes of MSCs in different scaffolds on day 3. Control-MSC and Control-MΦ were used as controls for comparison, respectively. Data presented as mean ± SD, n = 4 for each scaffold type, *p*-values are calculated using one-way ANOVA with Tukey correction, $*p < 0.05$, $**p < 0.01$, and $***p < 0.001$; ns, not statistically significant. The osteo-immune microenvironment created by MSCs co-cultured with macrophages at a cell ratio of 2:1 in the Tai Chi pattern was more conducive to osteogenic differentiation of MSCs than those created by MSCs to macrophages at 1:1, 1:2, 1:3, and 3:1 ratios, with widespread high expression of numerous osteogenic genes.

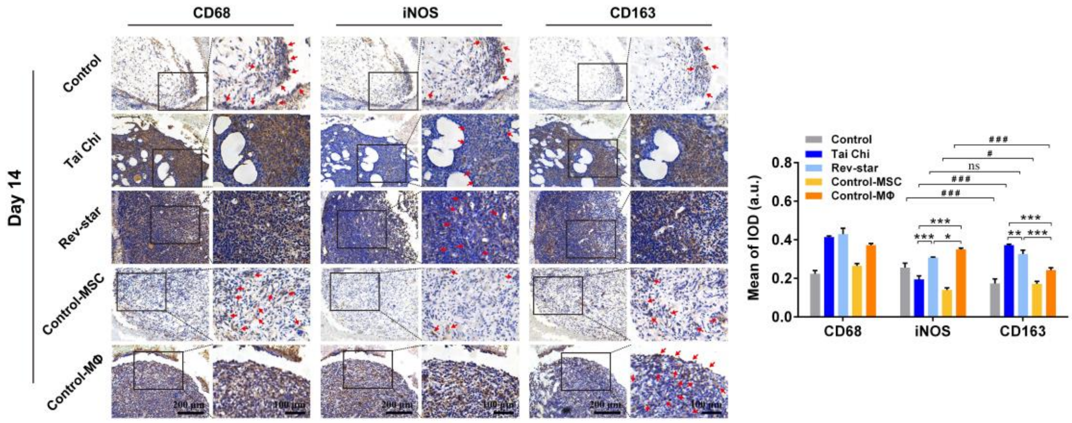

Figure S8. *In vivo* evaluation of macrophage polarization in multicellular patterning bioactive scaffolds after 14 days of subcutaneous implantation. Representative immunohistochemical staining images and quantitative analysis of macrophage polarization in different scaffold implants. Positive regions were labeled in brown. The red arrows representative positively stained cells. Data presented as mean ± SD, n = 3 for each scaffold type, *p*-values are calculated using one-way ANOVA with Tukey correction, *$p < 0.05$, **$p < 0.01$, and ***$p < 0.001$; ns, not statistically significant. At the ratio of MSCs to macrophages 2:1, the Tai Chi pattern in scaffolds activated anti-inflammatory M2 macrophage, providing a pro-healing microenvironment for tissue regeneration.

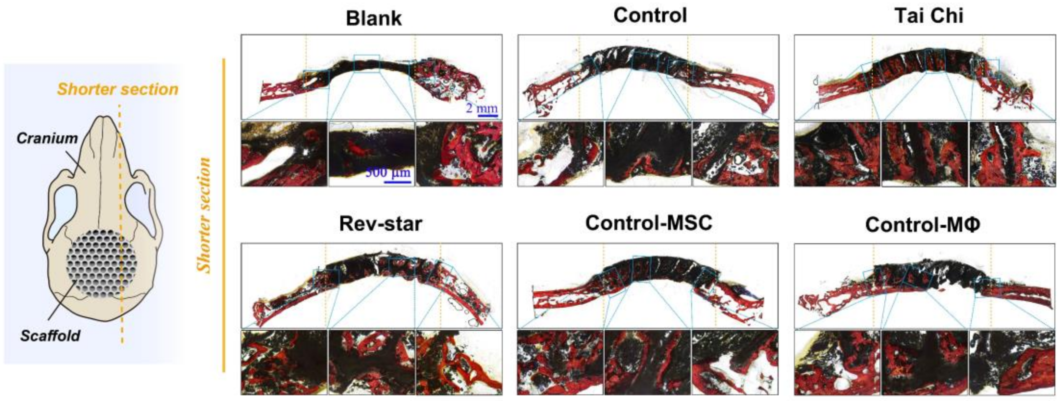

Figure S9. Evaluation of *in vivo* bone regeneration of multicellular patterning bioactive scaffolds after 12 weeks of implantation in rabbit cranial defects. Tissue sections with a short length perpendicular to the implant were prepared near the edge of the scaffolds and then used for histological staining analysis. The majority of the channels near the edges of multicellular patterning scaffolds were filled with newly formed bone tissue (red), indicating that the multicellular patterning scaffolds possessed enhanced osteogenic abilities.

Table S1 Sequences of real-time PCR primers

Primers	Sequences
CCR7	Forward 5'-CCATGACGGATACCTACCTGCT-3'<br>Reverse 5'-CCCTTACACAGGTAGACGCCAA-3'
CD206	Forward 5'-ATCCACGAGCAAATGTACCTCA-3'<br>Reverse 5'-TAGCCAGTTCAGATACCGGAA-3'
IL-1β	Forward 5'-CTACCTGTGTCTTTCCCGTG-3'<br>Reverse 5'-TTTGTTGTTCATCTCGGAGC-3'
Arg-1	Forward 5'-ATCAACACTCCCCTGACAACC-3'<br>Reverse 5'-TCGCAAGCCAATGTACACGAT-3'
iNOS	Forward 5'-ACGCTTCACTTCCAATGCAAC-3'<br>Reverse 5'-CAGCCTCATGGTAAACACGTTC-3'
IL-10	Forward 5'-GAGAAGCATGGCCCAGAAATC-3'<br>Reverse 5'-GAGAAATCGATGACAGCGCC-3'
IL-1rα	Forward 5'-AGAGCCCCTTATAGTCACGAA-3'<br>Reverse 5'-TACACCCTGCAAAAGTTGTTCC-3'
TNF-α	Forward 5'-CTGTAGCCCACGTCGTAGCAA-3'<br>Reverse 5'-TGTCTTTGAGATCCATGCCGTT-3'
VEGF	Forward 5'-GTCCCATGAAGTGATCAAGTTC-3'<br>Reverse 5'-TCTGCATGGTGATGTTGCTCTCTG-3'
CTSK	Forward 5'-GGCCAGTGTGGTTCCTGTTGG-3'<br>Reverse 5'-CCGCCTCCACAGCCATAATTCTC-3'
TRAP	Forward 5'-CACGATGCCAGCGACAAGAGG-3'<br>Reverse 5'-ATCTGTGCAGAGACGTTGCCAAG-3'
Wnt10b	Forward 5'-CGATACCCACAACCGCAACTCTG-3'<br>Reverse 5'-TCTCGCTCGCAGAAGTCAGGAG-3'
OSM	Forward 5'-TGCAGACACGGCTTCTAAGAACAC-3'<br>Reverse 5'-TGAGGAGCTGAGAGGAAGAGTTGG-3'
GAPDH	Forward 5'-AGAACATCATCCCTGCATCCAC-3'<br>Reverse 5'-TCAGATCCACGACGGACACA-3'
Runx2	Forward 5'-CCTCGAATGGCAGCACGCTA-3'<br>Reverse 5'-GCCGCCAAACAGACTCATCCA-3'
BMP-2	Forward 5'-TCCAGTCTTGCCGCCTCCAG-3'<br>Reverse 5'-CTTCGCCTCCTCCTCCTTCTCC-3'
ALP	Forward 5'-CACGGCGTCCATGAGCAGAAC-3'<br>Reverse 5'-CAGGCACAGTGGTCAAGGTTGG-3'
OCN	Forward 5'-AGACTCCGGCGCTACCTTGG-3'<br>Reverse 5'-CGGTCTTCAAGCCATACTGGTCTG-3'
OPN	Forward 5'-ATCTCCTTGCGCCACAGAATGC-3'<br>Reverse 5'-CGTCAGATTCATCCGAGTCCACAG-3'
OSX	Forward 5'-CCCTTCTCAAGCACCAATGG-3'<br>Reverse 5'-AGGGTGGGTAGTCATTTGCATAG-3'
BMPR1A	Forward 5'-TGCCCAGTGACCCATCCTATGAG-3'<br>Reverse 5'-TGTTCCAGCGGTTAGACACGATTG-3'

BMPR2	Forward 5'-TGCTGAGGAGAGGATGGCTGAAC-3'<br>Reverse 5'-AGTTGACATTGGGTTGACCGTTGG-3'
Smad1	Forward 5'-GCCACCATGAACTGAAGCCTCTG-3'<br>Reverse 5'-TACTCGCTGTGCCTCGGAACC-3'
Smad4	Forward 5'-CTGCCCTGTTGTGACTGTGGATG-3'<br>Reverse 5'-CTCGCTCTCTCAATCGCTTCTGTC-3'
Smad5	Forward 5'-AGCAGAGTACCACCGACAGGAC-3'<br>Reverse 5'-AGCGGAGAGCCCATCTGAGTAAG-3'
IL6ST	Forward 5'-GTGTGCTGAAGGAGGCGTGTC-3'<br>Reverse 5'-ACTGGACGTGGTTCTGTTGATGAC-3'
OSMR	Forward 5'-AAGCAGAAGGCAGCAAGACACAG-3'<br>Reverse 5'-TTGGCACGATTGGCGGCTTAG-3'
STAT3	Forward 5'-ACATCTGCCTGGACCGTCTGG-3'<br>Reverse 5'-AACAGCTCCACGATCCTCTCCTC-3'
β-catenin	Forward 5'-TGCCGTTCGCCTTCATTATGGAC-3'<br>Reverse 5'-TGGGCAAAGGGCAAGGTTTCG-3'
LRP5	Forward 5'-CTTCATCCACCGTGCCAACCTG-3'<br>Reverse 5'-TCTGCCAGTCTGTCCAGTAGAGTG-3'
Axin2	Forward 5'-CAGCAGTGTCATACCCTTCTTCCG-3'<br>Reverse 5'-GTGGTGGATGTAGTGGTGGTGAAC-3'
